# cuRnet: an R package for graph traversing on GPU

**DOI:** 10.1186/s12859-018-2310-3

**Published:** 2018-10-15

**Authors:** Vincenzo Bonnici, Federico Busato, Stefano Aldegheri, Murodzhon Akhmedov, Luciano Cascione, Alberto Arribas Carmena, Francesco Bertoni, Nicola Bombieri, Ivo Kwee, Rosalba Giugno

**Affiliations:** 1 Department of Computer Science, University of Verona, Strada le Grazie, 15, Italy, Verona, Italy; 2 Institute of Oncology Research (IOR), Via Vincenzo Vela 6, Bellinzona, Switzerland

**Keywords:** Graph traversal, GPU parallel implementation, Biological network analysis, High-throughput omics network annotation, Topological network analysis, Prize-collecting Steiner forest

## Abstract

**Background:**

R has become the de-facto reference analysis environment in Bioinformatics. Plenty of tools are available as packages that extend the R functionality, and many of them target the analysis of biological networks. Several algorithms for graphs, which are the most adopted mathematical representation of networks, are well-known examples of applications that require high-performance computing, and for which classic sequential implementations are becoming inappropriate. In this context, parallel approaches targeting GPU architectures are becoming pervasive to deal with the execution time constraints. Although R packages for parallel execution on GPUs are already available, none of them provides graph algorithms.

**Results:**

This work presents *cuRnet*, a R package that provides a parallel implementation for GPUs of the breath-first search (BFS), the single-source shortest paths (SSSP), and the strongly connected components (SCC) algorithms. The package allows offloading computing intensive applications to GPU devices for massively parallel computation and to speed up the runtime up to one order of magnitude with respect to the standard sequential computations on CPU. We have tested *cuRnet* on a benchmark of large protein interaction networks and for the interpretation of high-throughput omics data thought network analysis.

**Conclusions:**

*cuRnet* is a R package to speed up graph traversal and analysis through parallel computation on GPUs. We show the efficiency of *cuRnet* applied both to biological network analysis, which requires basic graph algorithms, and to complex existing procedures built upon such algorithms.

**Electronic supplementary material:**

The online version of this article (10.1186/s12859-018-2310-3) contains supplementary material, which is available to authorized users.

## Background

Biological networks are seen as graphs, where vertices represent elements and edges are the relationships among them. Analyzing biological networks mostly means applying basic graph traversal algorithms to find, for instance, how two vertices are connected, which vertices can be reached by a source, and which part of the network is highly interconnected, i.e., every vertex is reachable from every other vertex. These tasks are commonly embedded in more crucial sophisticated analyses [1, 2] to predict, for example, protein functions [3] or to study complex diseases by relating protein interaction networks to specific conditions [4–7]. Due to the constantly increasing data set complexity, such applications require high-performance algorithms, for which classic sequential implementations are become inappropriate. Alternative solutions are given by parallel approaches, and in particular by those based on GPU architectures, which allow sensibly reducing the algorithm execution time [8].

In the context of biological network analysis and, more in general, for statistical computing in Bioinformatics, R is becoming one of the most widely used programming environment. It provides easy-to-use packages to programmers and analysts for efficient and flexible data modeling and analysis [9]. In this context, even though some R packages based on GPU kernels have been proposed (e.g., *gpuR* for algebraic operations https://cran.r-project.org/package=gpuR), none of them provides parallel implementations of algorithms for network analysis.

This work presents *cuRnet*, an R package that provides a wrap of parallel graph algorithms to the R environment. As an initial proof of concept, *cuRnet* includes basic data structures for representing graphs, a parallel implementation of Breadth-First Search (BFS) [10], Single Source Shortest Paths (SSSP) [11], and Strongly Connected Components (SCC) [12]. The package makes available GPU solutions to R end-users in a transparent way, such that GPU modules are invoked by R functions.

*cuRnet* has been compared with the BFS, SSSP, and SCC implementation of the iGraph R package (http://igraph.org/r/). Tests were run over on annotated undirected protein interaction networks and on directed homology networks provided by the STRINGdb [13].

*cuRnet* outperformed the iGraph sequential algorithms especially on the largest networks. An average speed-up of 3 × have been observed, with a maximum of 30 ×.

*cuRnet* SCC and SSSP were used to underscore their ability in helping researchers in providing clues on putative functional context of ncRNA molecules, and guide the selection of a relevant functional readout [14, 15]. For this aim, we used available RNA sequencing dataset of 21 prostate cancer cell lines (GEO accession number GSE25183) to predict coexpression networks. We also show how enabling the GPU implementation of graph traversal algorithms in R has a potential to speed up existing complex procedures whose implementation mainly depends on such calculations. The PCSF package for R [16] is an example, which solves the Prize-collecting Steiner Forest problem by making a massive use of SSSP. It performs user-friendly analysis of high-throughput data using the interaction networks (protein-protein, protein-metabolite or any other type of correlation-based interaction networks) as a template. It interprets the biological landscape of interactome with respect to the data, i.e., to detect high-scoring neighbourhoods to identify functional modules. A real case application of intensive PCSF computation is reported on the analysis of Diffuse large B-cell lymphoma gene expression data.

*cuRnet* and the PCSF application accelerated with *cuRnet* are freely available on https://bitbucket.org/curnet/curnet.

## Methods

**Fig. 1:**
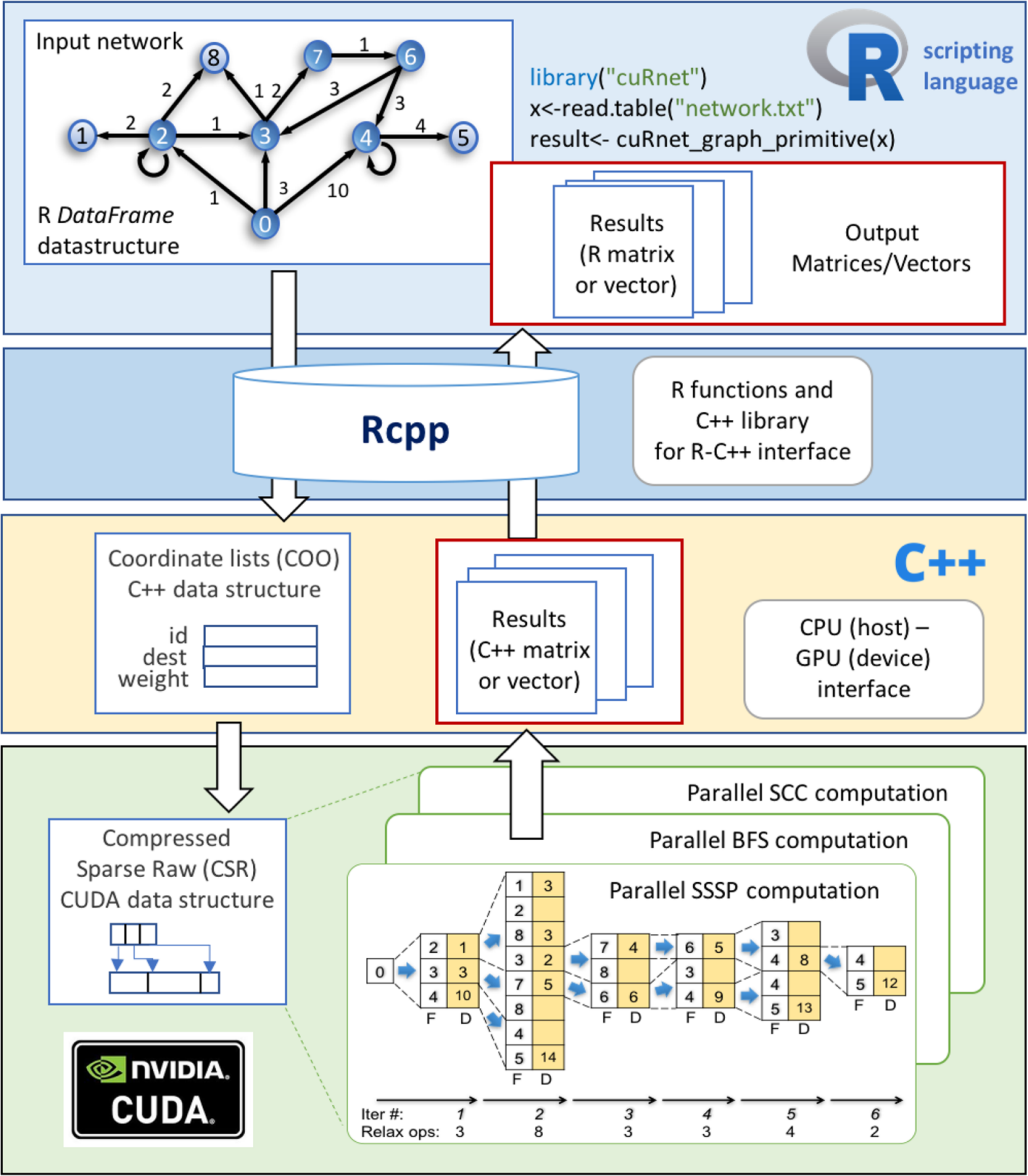
*cuRnet* stack overview *cuRnet* stack overview

Figure 1 shows an overview of the full *cuRnet* stack, by which R data is passed, as input data, to the GPU environment for parallel computation. The input network is represented, in R, through a standard R data frame, where every edge between two vertices is stored with the corresponding weight. By exploiting the *Rcpp* library of R, an R-C++ wrapper has been developed to automatically translate the network from the standard R representation to a C++ data structure, and to link the algorithm invocation from the R to the C++ environment.

The network representation in the C++ environment relies on the coordinate list (COO) data structure, which is a mandatory step to generate the compressed sparse row (CSR) data structure for the GPU computation. CSR is a well-known storage format to efficiently represent graphs, and it allows reaching high performance during the graph traversal on the GPU.

The C++ interface allows handling the interaction with the GPU device. It generates the host (CPU) representation of the graph starting from the rows in the data frame, it initializes the GPU kernel, it handles the host (CPU)-device (GPU) data exchanging, and, finally, it runs the kernel for the parallel computation. The computation result is retrieved from the device and passed back to R through the Rcpp/C++ layers.

In what follows we briefly describe the parallel graph traversal algorithms implemented in *cuRnet*. Given a graph *G*(*V*,*E*), with a set *V* of vertices, a set *E* of edges, and a weight function $w: E \to \mathbb {R} $, *cuRnet* takes *G* in a dataframe X having three columns listing the network edges and their weights. The dataframe can be built from an iGraph object or from a textual file (.csv). The following lines invoke the loading of the *cuRnet* package and the construction of the graph data structure:


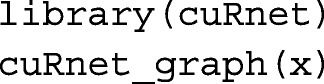


We refer the reader to (https://bitbucket.org/curnet/curnet) for a complete manual of the *cuRnet* usage.

### Parallel implementation of breadth-first search for GPUs

The parallel graph traversal through BFS [10], which is listed and analyzed in Additional file 1: Section 1 -Algorithm 1 and Figure S1, respectively, explores the reachable vertices, level-by-level, starting from a source *s*. *cuRnet* implements the concept of frontier [17] to achieve work efficiency. A frontier holds all and only the vertices visited at each level. The algorithm checks every neighbour of a frontier vertex to see whether it has been already visited. If not, the neighbour is added into a new frontier. *cuRnet* implements a frontier propagation step through two data structures, *F*_1_ and *F*_2_. *F*_1_ represents the actual frontier, which is read by the parallel threads to start the propagation step. *F*_2_ is written by the threads to generate the frontier for the next BFS step. At each step, *F*_2_ is filtered and swapped into *F*_1_ for the next iteration. When a thread visits an already visited neighbour, that neighbour is eliminated from the frontier. When more threads visit the same neighbour in the same propagation step, they generate duplicate vertices in the frontier. *cuRnet* implements efficient duplicate detection and correction strategies based on hash tables, advanced strategies for coalesced memory accesses, and warp shuffle instructions. Moreover, it implements different strategies to deal with the potential workload imbalance and thread divergence caused by any actual biological network non-homogeneity. These include prefix-sum procedures to efficiently handle frontiers, dynamic virtual warps, dynamic parallelism, multiple CUDA kernels, and techniques for coalesced memory accesses.

The BFS result is a matrix *s*×|*V*|, where *s* is the number of vertex sources from which the BFS is run. Each entry in the matrix is the depth of the BFS from a source to a graph vertex. The matrix is retrieved from the GPU device to R through the Rcpp/C++ layers. BFS is ran by invoking the following *cuRnet* function in the R environment:


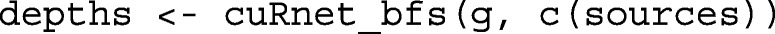


### Parallel implementation of single-source-shortest-path for GPU

The *cuRnet* CUDA implementation of the SSSP algorithm is based on the Bellman-Ford’s approach [11]. The parallel algorithm is reported in Additional file 1: Section 1. *cuRnet* SSSP visits the graph and finds the shortest path *d* to reach every vertex of *V* from source *s*. Also in this case, *cuRnet* exploits the concept of frontier to deal with the most expensive step of the algorithm (i.e., the relax procedure). At each iteration *i*, the algorithm extracts, in parallel, the vertices from one frontier and inserts the active neighbours in the second frontier for the next iteration step. Each iteration concludes by swapping the contents of the second frontier (which will be the actual frontier at the next iteration) into the first one. Indeed, the frontiers allow working only on active vertices, i.e., all and only vertices whose tentative distance has been modified and, thus, that must be considered for the relax procedure at the next iteration.

The result is a double numeric matrix (i.e., *distances* and *predecessors*), which are retrieved from the GPU device to R through the Rcpp/C++ layer. They are obtained by invoking the *cuRnet* functions CURNET_SSSP and CURNET_SSSP_DISTS for the matrix of shortest paths (returned as lists of predecessor vertices) and the corresponding source-destination distances:


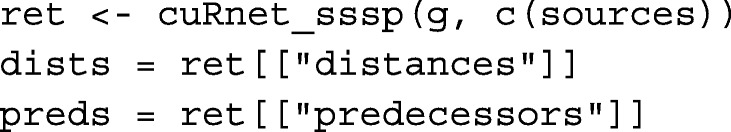


### Parallel implementation of strongly-connected components for GPU

*cuRnet* implements a multi-step approach that applies different GPU-accelerated algorithms for SCC decomposition [12]. The algorithm is reported in Additional file 1: Section 1. The multi-step approach consists of 3 phases. In the first phase it iterates a trimming procedure to identify and delete vertices of *G* that form trivial SCCs (i.e., vertices with no active successors or predecessors). In the second phase it iterates a forward-backward algorithm to identify the main components. The first step is related to the choice of the pivot for each set, where heuristics can be applied to maximize vertices coverage within a single iteration. Forward and backward closure is then computed from this vertex, and up to four subgraphs are generated. The first one is the component which the pivot belongs to, and it is calculated as the intersection of the forward and backward closure. The other three sets are SCC-closed subgraphs that can be processed in parallel at the next iteration. They correspond to the non-visited vertices in the current set, to the forward closure but not to the backward one, and to the backward-reachable vertices, respectively. In the third phase the approach runs a *coloring* algorithm to decompose the rest of the graph. A unique color is firstly assigned to each vertex. The max color is then propagated to the successor non-eliminated vertices until no more updates are possible. Pivots are chosen as the vertices which color is unchanged. Running the backward closure from these vertices on the corresponding set, *cuRnet* detects the components labelled with that color.

The *cuRnet* SCC computation results in a vector of associations between vertices and strongly component IDs. It is retrieved from the GPU device to R through the Rcpp/C++ layer and obtained by invoking the following *cuRnet* function:


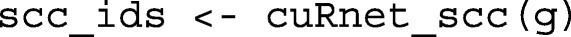


## Results

We evaluated the *cuRnet* performance by comparing its execution time with the corresponding sequential implementations provided in the *iGraph* R package (http://igraph.org/r/). The *cuRnet* software requires a GPU device with compute capabilities at least 3.0. We performed tests on two different GPU devices running on a machine equipped with an AMD Phenom II X6 (3GHz) host processor, 64 GB RAM, Ubuntu 14.04 OS, and CUDA Toolkit v 8.0. The first device is an NVIDIA Maxwell GeForce GTX 980 GPU having 16 SMs (2048 CUDA cores) and 8 GB of GDDR5 memory, and it is capable of concurrently executing 32,768 threads. The second device is an NVIDIA Tesla K40 comprised of 12 GB of GDDR5 memory and 15 SMs (2880 CUDA cores), and it is able of concurrently executing 30,720 threads The two GPU devices have equal memory technology but they differ in the number of threads that they can concurrently execute and in the internal architecture. The technology of the Maxwell architecture is more recent than the Tesla one. For these reasons, the first device is expected to show better performances, compared with the second device, in many applications. In what follows, we show the main results we obtained by running tests on the Maxwell device, while we run a subset of the benchmarks on the Tesla device to show a comparison of performance between the two architectures.

### Data

We used the STRING dataset [13], which mainly contains Protein-Protein Interaction (PPI) networks of several organisms, varying from microbes to eukaryotes. We used the R package STRINGdb to download the data. We refer the reader to Additional file 1: Section 2 for details on the data.

We retrieved the *undirected unlabeled networks* related to *Homo sapiens*, *Danio rerio* and *Zea mais* (see Additional file 1: Figures S2, S3 and S4 for a description of the network characteristics). Those species were chosen among the organisms having the largest networks stored in STRING, to cover the biological diversity that can be encountered in performing analysis of biological networks. For each network, we varied the threshold on the assigned edge scores to obtain sparse as well as dense networks.

We created a benchmark of *undirected label networks* by using the pvalues of differential expression values regarding the treatment of A549 lung cancer cells by means of Resveratrol, a natural phytoestrogen found in red wine and a variety of plants shown to have protective effects against the disease [13] (see Additional file 1: Figure S5). We used such values to label the above networks.

We also created a set of *directed unlabelled networks* (see Additional file 1: Figure S6) as follows. We used the complete set of 115 archaea species to create homology networks having incremental amount of involved organisms. The homology information between proteins is measured by sequence BLAST alignments. For each protein, STRING reports the best BLAST hits [18], w.r.t. the given species. Horizontal gene transfer is a frequent phenomenon in microbes [19], and homology networks are used to search for gene families shared by several organisms [20].

The running time to create graph data structures in *cuRnet* and iGraph from the above datasets is reported in Additional file 1: Figures S7 and S8. In general, *cuRnet* requires half the time of iGraph to perform such a task.

### *cuRnet* performance

**Fig. 2:**
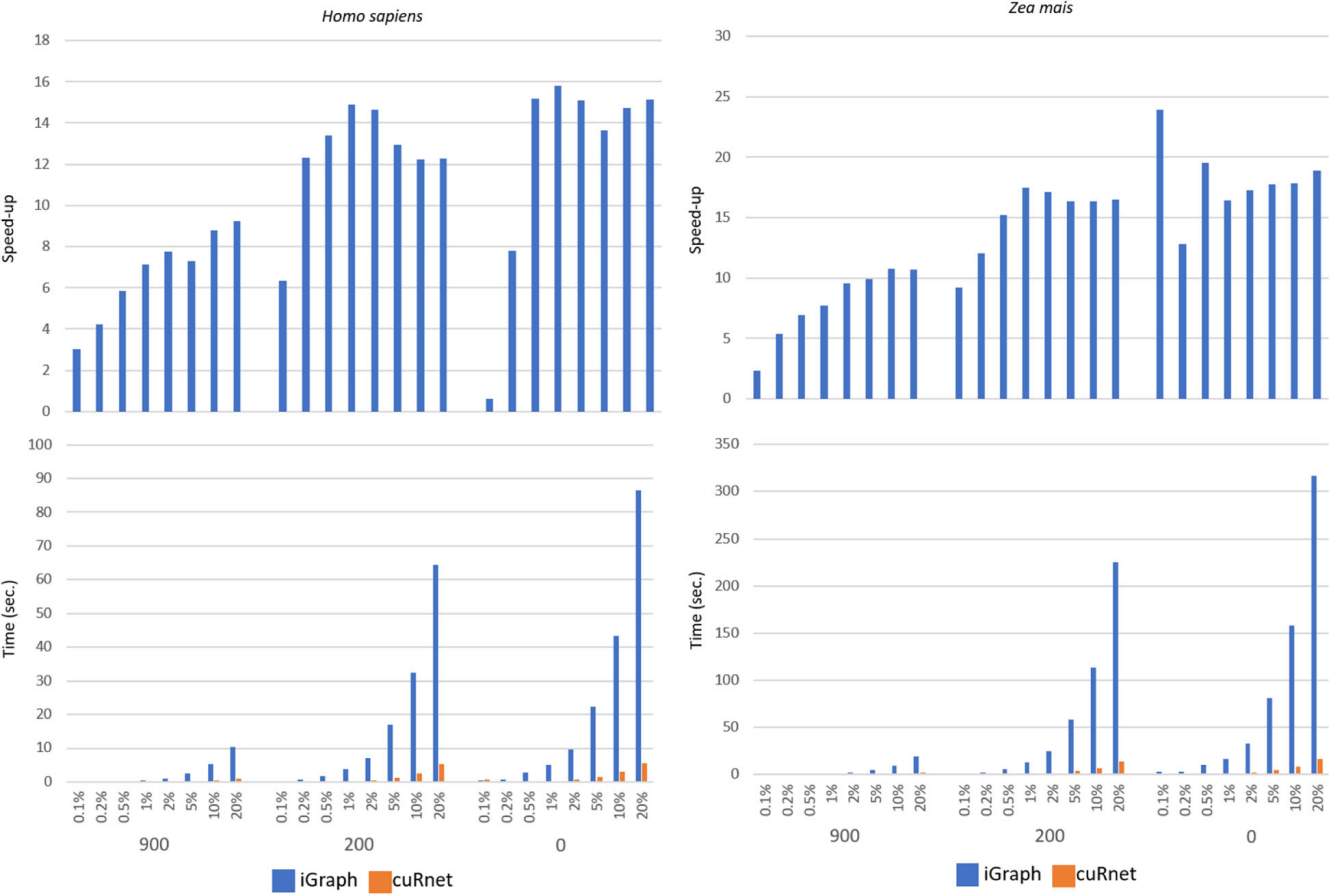
*cuRnet* performance vs iGraph on computing breath first search. Three different score thresholds, 0, 200 and 900, were applied, and different amounts of source vertices were selected

**Fig. 3:**
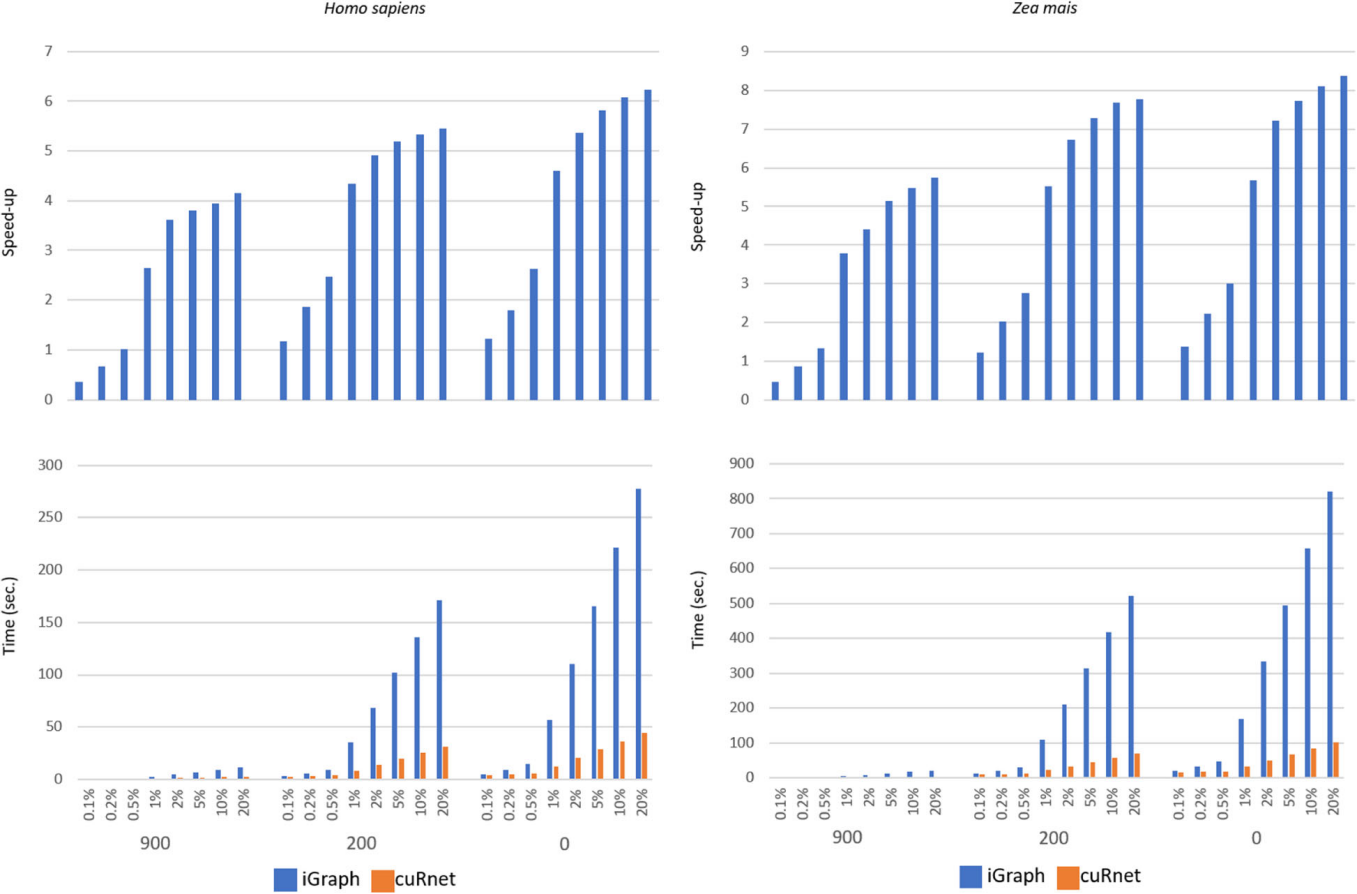
*cuRnet* performance vs iGraph on computing shortest paths distances. Three different score thresholds, 0, 200 and 900, were applied, and different amounts of source vertices were selected. The underlying charts show running times of *cuRnet* and iGraph in calculating distance of shortest paths within the PPI of the selected species for every combination of score threshold and amount of selected sources

We tested *cuRnet* BFS on undirected unlabeled networks and SSSP on undirected labeled networks related to *Homo sapiens*, *Danio rerio* and *Zea mais* by varying the number of sources ranging from just to few vertices to a 20% of vertices. Figures 2 and 3 (see also Additional file 1: Figures S9 and S10) show the execution time of the BFS and SSSP, as well as the corresponding speedup w.r.t. the sequential counterpart. Running times were evaluated as an average of 10 runs.

Additional file 1: Figures S11, S12, S13 and S14 show the total running time including the call to the function primitives, plus the time required for building the graph data structures. Highly functional networks have small sizes and the execution time of the two implementations is in terms of few seconds, obtaining however speedups up to 5 ×. The time of both packages highly depends on the number of source vertices, but the slope of *cuRnet* is sensibly lower than iGraph. On average, iGraph shows similar performance up to a small percentage of sources (0.5%). Above that, *cuRnet* shows up to 15 × speedup w.r.t. the sequential counterpart. The time requirements and the general speedup are similar for the three species.

**Fig. 4:**
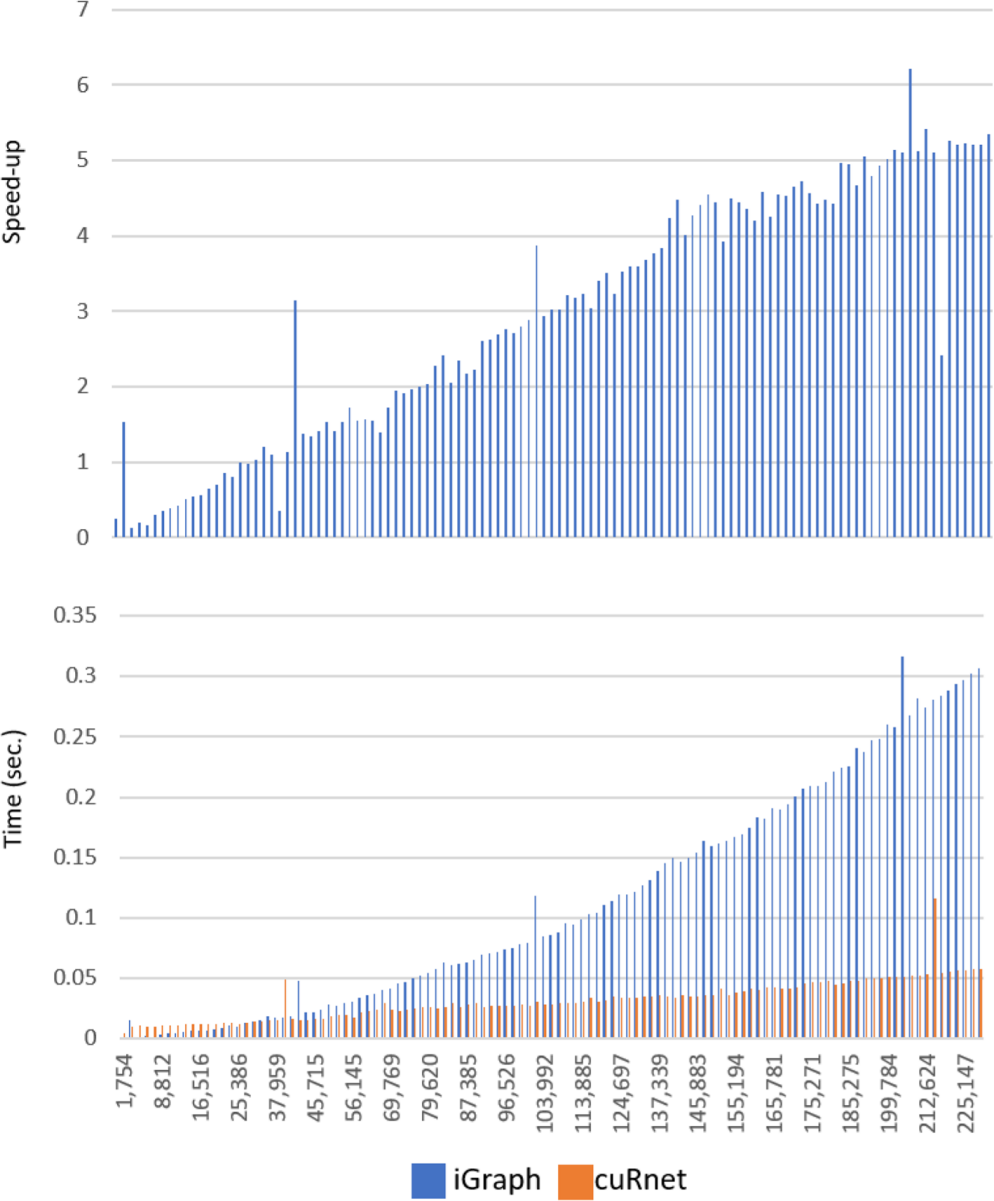
*cuRnet* performance vs iGraph on computing strongly connected components. Running times, and corresponding speed-ups, of *cuRnet* and iGraph on increasing the size of the extracted homology network, up to the final one of 114 species. Left-side charts show total running, includes the call to the SCC primitive, plus the time required for construction of graph data structures. Right-size charts show comparisons performed by timing only the execution of the SCC algorithm

We tested *cuRnet* SCC performance on directed unlabelled networks representing inter-species proteins homology. Figure 4 shows the running time and corresponding speed-ups by increasing the size of the extracted homology networks, up to the final one of 114 species. As for the previous benchmarks, running times were evaluated as an average of 10 runs. Additional file 1: Figure S15 reports the total running time including the graph data structure generation. *cuRnet* shows an extremely low slope w.r.t. iGraph, and the speedup increases by increasing the network size up to a maximum of 14 ×. Additional file 1: Figures S16, S17 and S18 report the performance of *cuRnet* measured by running the software on two different GPU architectures. Regarding BFS, the device with the Maxwell architecture outperforms the Tesla device, however also the less recent device shows good speed-ups, up to 10 ×, w.r.t. iGraph.

**Fig. 5:**
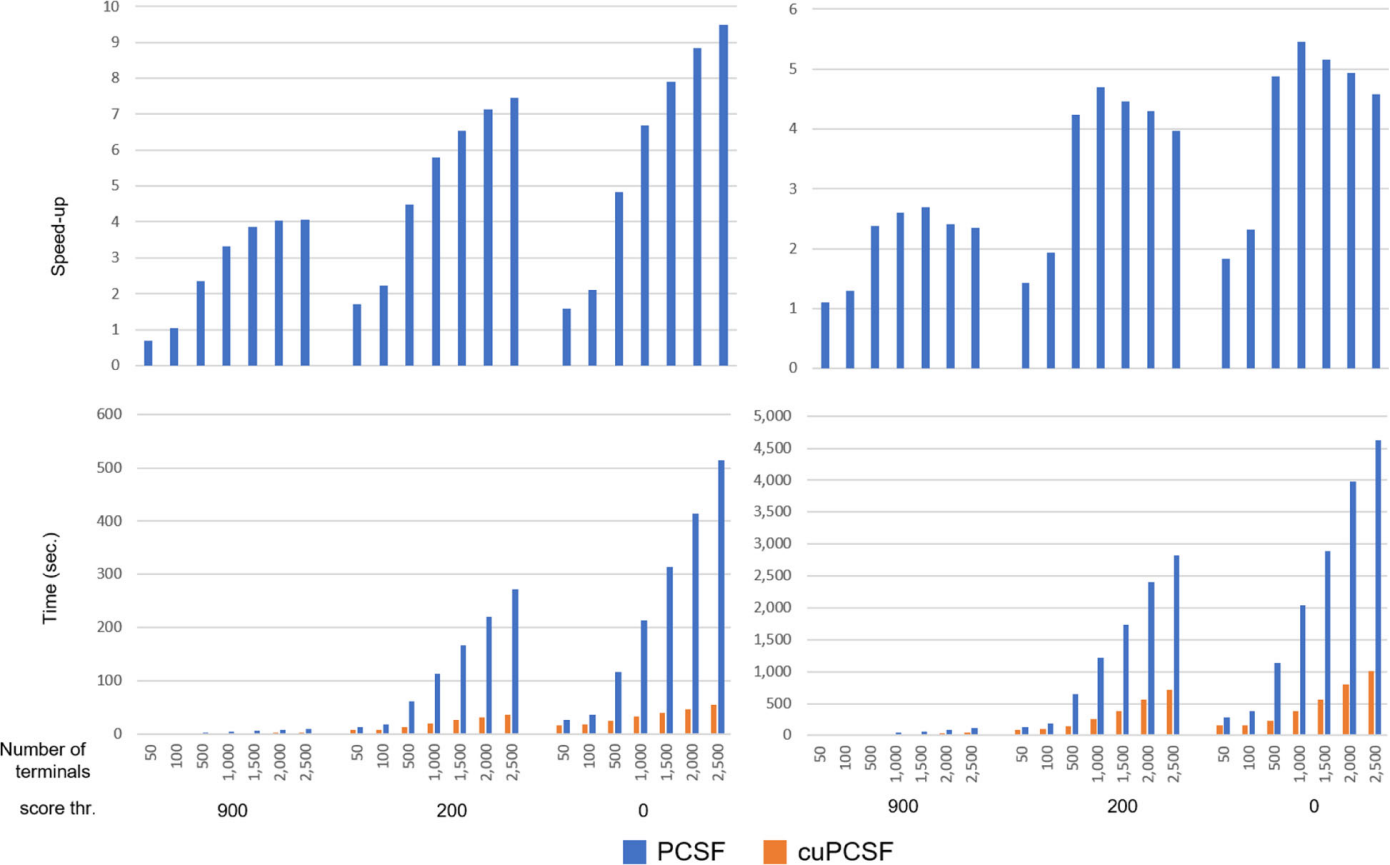
Performance of the GP-GPU aided PCSF package versus its serial counterpart. Charts show running time and related speed-ups of the original PCSF R package and the modified version where the SSSP primitive of the Boost library has been replaced with the GP-GPU based approach, named cuPCSF. Tests were performed on the human direct label PPI network by applying three score thresholds. Right-side charts show performances for a single PCSF run, while charts on the right side show executions of randomized selections. The GP-PGU based PCSF reaches speed-ups up to 9 ×. The parallelized version outperforms better on increasing the network size as well as the amount of terminal vertices. Randomization procedures introduce additional non-parallelized steps performed by the methodology, thus speed-ups reach a maximum of 5 ×

Finally, we tested a modified version of PCSF R package [16] where the original sequential SSSP implementation has been replaced by the parallel SSSP implementation of *cuRnet*. PCSF, taken an input network, may give prizes to vertices according to the measurements of differential expression, copy number, or number of gene mutations. After scoring the interactome, the PCSF identifies high-confidence subnetworks, the neighborhoods in interaction networks potentially belonging to the key pathways that are altered in a disease. It also interactively visualizes the resulting subnetworks with functional enrichment analysis. The running time of the PCSF module is highly dominated by SSSP computations and the application of the *cuRnet* SSSP provided up to 9 × speedup for the total execution times of the PCSF (see Fig. 5). This allows for even more rigorous computations on larger networks.

## Discussion

*cuRnet* allows users to quickly retrieve ncRNA-pathway associations and individual genes contributing to them. To evaluate the *cuRnet* performance in making highly confident ncRNA function predictions, we analysed a case study with the well-known lncRNA involved in cancer called MALAT1. Noncoding RNAs (ncRNAs) are emerging as key molecules in human cancer but only a small number of them has been functionally annotated [15]. Using the guilt-by-association principle is possible to infer functions of lncRNAs on a genome-wide scale [21]. This approach identifies protein coding genes significantly correlated with a given lncRNA using gene-expression analysis. In combination with enrichment strategies, it projects functional protein coding gene sets onto mRNAs correlated with the lncRNA of interest, generating hypotheses for functions and potential regulators of the candidate lncRNA. We used a public RNA sequencing dataset of 21 prostate cancer cell lines sequenced on the Illumina Genome Analyzer and GAII (GEO accession number GSE25183) and built up a large-scale gene association network using *cuRnet* SCC (Pearson method as pairwise correlations). We extracted the sub-networks where MALAT1 is present and calculated single-source shortest paths, mean distance of shortest paths within this subnetwork, and mean distance of shortest paths over the whole big graph. Gene Set Enrichment Analysis (GSEA) was carried out to identify associated biological processes and signalling pathways [22]. We computed overlaps of genes in the MALAT1 sub-networks with gene sets in MSigDB C2 CP (Canonical pathways) and hallmark gene sets [22]. Several cancer related pathways such as epithelial mesenchymal transition (EMT) and DNA replication were enriched, which implies that MALAT1 sub-networks might be involved in the metastasis related pathways [23]. In addition, we identified an over-representation of gene sets that corresponds to the validated MALAT1 functionality reported in the literature: cell cycle, e2f-targets, proliferation, B-MYB-related, and G2M checkpoint [14, 24].

We applied the PCSF to analyze Diffuse large B-cell lymphoma (DLBCL), which is the most common form of human lymphoma. Based on gene expression profiling studies DLBCL can be divided into two subgroups, the germinal center B-cell (GCB) and the activated B-cell like (ABC), with different clinical outcome and response to therapies [25, 26]. Therefore, it is important to understand underlying molecular mechanism of two subtypes. A public gene expression datasets GSE10846 from Gene Expression Omnibus online repository (https://www.ncbi.nlm.nih.gov/geo) has been used in the analysis. The dataset is composed of 350 patients being 167 ABC and 183 GCB. We run the PCSF separately for ABC and GCB patients providing top 100 differentially expressed genes as terminals and their absolute fold changes as prizes. The STRING database (version 13) [27] is provided as a template network by applying some filtering steps described in [6], which afterwards had 15,405 nodes and 175,821 genes.

**Fig. 6:**
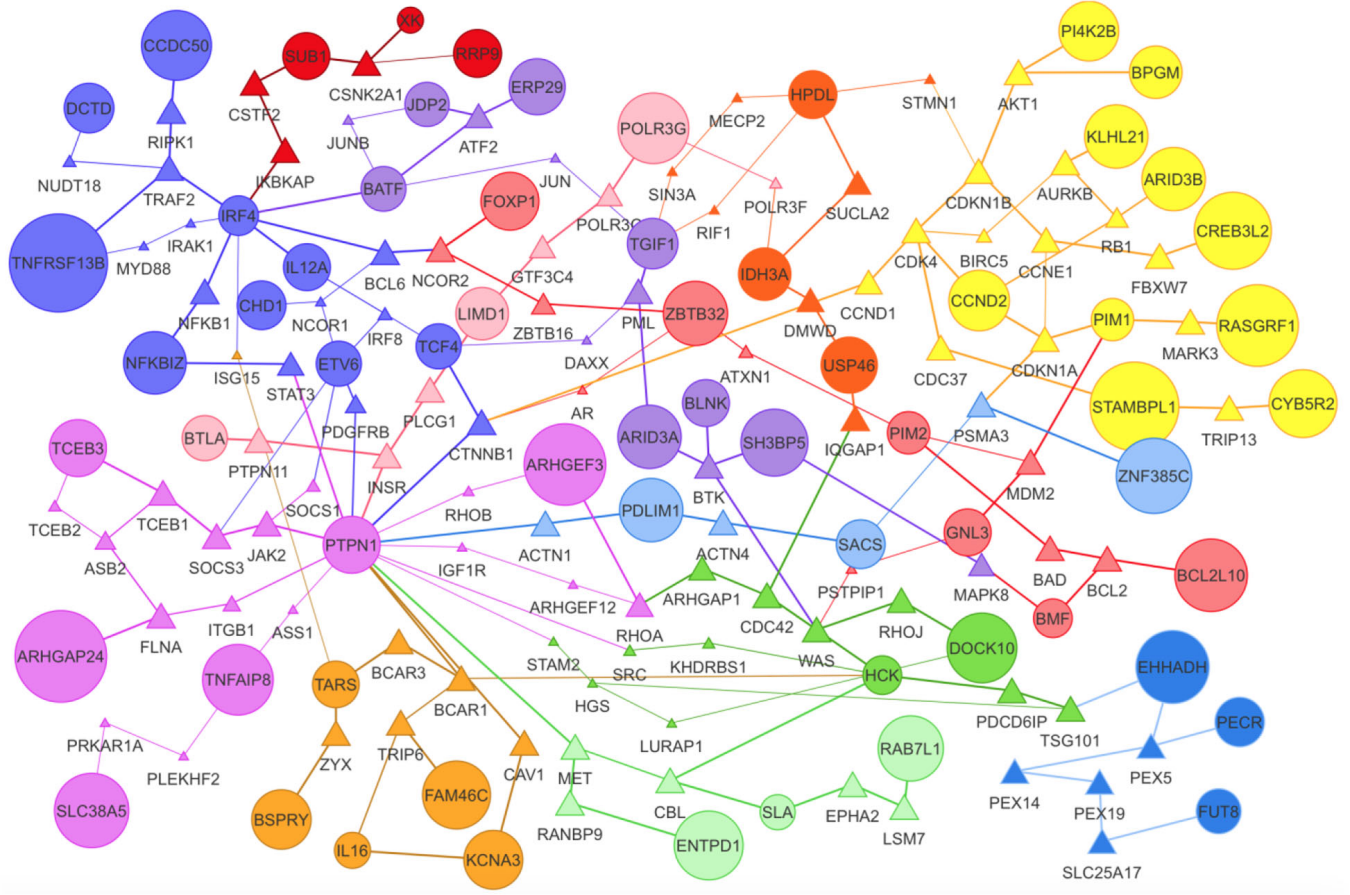
The PCSF subnetworks for ABC patients. The node sizes and edge widths are proportional to the number of appearance in multiple PCSF runs. Circular nodes are terminals and algorithm uses triangular nodes to connect terminals. Nodes are colored according to subnetwork membership. The resulting subnetwork for ABC patents was significantly enriched in NFKB pathway (cluster in purple located at top right of the figure) and composed of up-regulated ABC genes including IRF4, FOXP1, IL6, BATF and PIM2

An interactive visualization of the subnetwork for ABC patients is shown in Fig. 6. PCSF also performs enrichment analysis on subnetworks by employing either EnrichR [28] API or topGO [29] that can be specified by the user. For the resulting subnetwork of ABC patients, the hallmark of ABC-DLBCL, as constitutive activation of nuclear factor kappa-B (NFKB) signalling, was confirmed by the enrichment of NFKB pathway (cluster in purple) and up-regulation of well defined ABC genes including IRF4, FOXP1, IL6, BATF and PIM2 among others [30]. In parallel, PCSF subnetwork for GCB patients (see Additional file 1: Figure S19) showed activation of the PI3K/Akt/mTOR signalling pathway (cluster in red) and over-expression of germinal center markers such as BCL6, LMO2, MME (CD10) and MYBL1, reproducing the findings given in [30, 31].

## Conclusion

*cuRnet* has been developed to be easy to use both as a stand-alone analysis application and as a core primitive to be incorporated in more complex algorithmic frameworks. *cuRnet* has been structured to modularly include, as current and future work, a wide collection of algorithms for biological network analysis.

## Additional file

Additional file 1 Supplemental materials. (PDF 1695 kb)

## Abbreviations

BFS: Breadth-first search
GPU: Graphic processing unit
PCSF: Prize-collecting steiner forest
SCC: Strongly connected component
SSSP: Single source shortest path
