# Correction to: cuRnet: an R package for graph traversing on GPU

**DOI:** 10.1186/s12859-018-2484-8

**Published:** 2018-11-27

**Authors:** Vincenzo Bonnici, Federico Busato, Stefano Aldegheri, Murodzhon Akhmedov, Luciano Cascione, Alberto Arribas Carmena, Francesco Bertoni, Nicola Bombieri, Ivo Kwee, Rosalba Giugno

**Affiliations:** 1 Department of Computer Science, University of Verona, Strada le Grazie, 15, Verona, Italy; 2 Institute of Oncology Research (IOR), Via Vincenzo Vela 6, Bellinzona, Switzerland

## Correction

After publication of this supplement article [1], it was brought to our attention that reference 10 and reference 12 in the article are incorrect.

As such, please be advised that the correct versions of these references are:
