# Supplementary material for: cuRnet: an R package for graph traversing on GPU
Source: BMC Bioinformatics. 2018 Oct 15;19(Suppl 10):356. doi: 10.1186/s12859-018-2310-3 (PMC6191969; doi:10.1186/s12859-018-2310-3)
Supplement: Supplementary file 1 — Supplemental materials. (PDF 1695 kb) [file 12859_2018_2310_MOESM1_ESM.pdf]

RESEARCH

Supplemental materials of cuRnet: an R package for graph traversing on GPU

Vincenzo Bonnici[1], Federico Busato[1], Stefano Aldegheri[1], Murodzhon Akhmedov[2], Luciano Cascione[2], Alberto Arribas Carmena[2], Francesco Bertoni[2], Nicola Bombieri[1], Ivo Kwee[2] and Rosalba Giugno[1*]

*Correspondence: rosalba.giugno@univr.it
[1]Department of Computer Science, Strada le Grazie, 15, Italy, Verona, Italy
Full list of author information is available at the end of the article

Graph traversal algorithms for GPU

Algorithm 1 describes the parallel BFS algorithm implemented in *cuRnet* [1]. It explores the reachable vertices, level-by-level, starting from a source vertex s.

Algorithm 1 Parallel breath-first search algorithm

1: **for all** vertices $u \in V(G)$ **do**
2:     $d(u) = \infty$
3: $d(s) = 0$, $F_1 = s$, $F_2 = \emptyset$
4: level = 1
5: **while** $F_1 \neq \emptyset$ **do**
6:     **parallel for** vertices $u \in F_1$ **do**
7:         $u \leftarrow$ DEQUEUE(F_1)
8:         **parallel for** vertices $v \in adj\,[u]$ **do**
9:             **if** $d(u) = \infty$ **then**
10:                 $d(u) = level$
11:                 ENQUEUE(F_2, u)
12:         **end**
13:     **end**
14:     level = level + 1
15:     SWAP(F_1, F_2)
16:     $F_2 = \emptyset$
17: **end**

We selected the BFS for GPUs proposed in [1] (*BFS-4K*) since it is actually the main efficient BFS implementation at the state of the art. Figure 1 shows the results of a comparison we performed between *BFS-4K*, *Gunrock* [2], and *B40C* [3]. The network dataset has been composed by selecting different graphs from the University of Florida Sparse Matrix Collection [4], the 10th DIMACS Challenge [5], and the SNAP dataset [6]. It is important to note that this work targets many-core architectures (GPUs) as, for graph traversing, they allow reaching higher speedup w.r.t. multi-core architectures at low cost [1],[2],[3]. Indeed, *cuRnet* can be used in any HW/SW architecture with a standard and cheap graphics processing unit (e.g., NVIDIA GPU). Our future work consists of extending the acceleration concept to other more complex, high-end architectures (e.g., multi-node GPUs or multi-node many-cores like that adopted in [7]). The parallel SSSP algorithm implemented in *cuRnet* is based on the Bellman-Ford's approach [8]. It can be derived from Algorithm 1 by replacing lines 8-12 with the following lines:

parallel for vertices $v \in adj\,[u]$ **do**
    if $d(u) + w < d(v)$ **then** // *relax procedure*
        $d(v) = d(u) + w$
        ENQUEUE(F_2, v)

end

end

Finally, the parallel SCC algorithm implemented in *cuRnet* is described in Algorithm 2. It implements a multi-step approach that applies different GPU-accelerated algorithms for SCC decomposition[9].

Data

We used the STRING dataset [10], which mainly contains Protein-Protein Interaction (PPI) networks of several organisms, varying from microbes to eukaryotes. We used the R package STRINGdb. We retrieved the undirected networks related to *Homo sapiens*, *Danio rerio*, and *Zea mais* (Figures 2, 3 and 4 show their properties). These three organisms belong to the set of core species of STRING. This guarantees a good reliability of them. The human PPI is the smallest one, with 19k vetices and 11M edges, and the Maize's network is the largest one, with 33k vertices and 34M edges. The networks show differences in the degree distribution, as well as in the distribution of the STRING score assigned to their edges. STRING assigns to each interaction a functional score, combining multiple information, ranging from 0 to 1000. Thus, in addition to the complete PPIs, supplementary networks were extracted by applying thresholds to edge scores. A threshold on the value 900 has been fixed to discard edges with lower score producing a sparse network of highly functional connections [10]. An intermediate threshold on the value 200 has been fixed to remove low significant predicted interactions.

The STRING package provides a real-case example reporting differential expression values regarding the treatment of A549 lung cancer cells by means of Resveratrol, a natural phytoestrogen found in red wine and a variety of plants shown to have protective effects against the disease. The example is referred to the GEO (Gene Expression Omnibus) GSE9008 study. We used such data to label the above described PPI networks according to the pvalues (see Figure 5).

Figure 6 reports the properties of a dataset of direct unlabelled homology networks built on the complete set of 115 archaea species from STRINGdb. The homology information between proteins is measured by sequence BLAST alignments. For each protein, STRING reports the best BLAST hits, w.r.t. a given species. The number of edges (best hists) increases with the number of vertices (proteins), and about 75% of edges represents bidirectional hits. The final network, composed by the proteins of all the 114 species, has 229k vertices and more than 9M edges. Degree distributions show a prevalence for low values, that indicates the presence of strain-specific genes. The outgoing degree distribution is bounded by the amount of 114 species, but much higher connectivity is shown for incoming degrees.

Performance

Figures 7 and 8 report the running time to create the graph data structures from the R data. They show that *cuRnet* requires half the time w.r.t. iGraph to accomplish such a task. Figures 9 and 10 show running times regarding the computation of BFS and SSSP on the Danio rerio PPI. Figures 11, 12, 13, 14, and 15 compare the running time to compute BFS, SSSP and SCC including the time to build the graph data structures. *cuRnet* outperforms iGraph confirming the speedups reported in

Algorithm 2 Parametric Multi-Step SCC Decomposition algorithm

```
F_1 ← V(G), F_2 ← ∅, P_1 ← ∅, P_2 ← ∅
parallel for vertices u ∈ V(G) do
    color(u) ← undefined
end

t ← 1
trimmable ← true
while F_1 ≠ ∅ ∧ t ≤ MAX_TRIM ∧ trimmable do
    trimmable ← false
    parallel for vertices u ∈ F_1 do
        if TRIM(F_1, u) = false then
            INSERT(F_2, u)
        else
            color(u) ← u
            trimmable ← true
        end
    end
    F_1 ← F_1 \ F_2
    t ← t + 1
end

INSERT(P_1, F_1)
fb ← 1
while P_1 ≠ ∅ ∧ fb ≤ MAX_FB do
    parallel for set S ∈ P_1 do
        p ← PIVOTSELECTION(S)
        F ← FWD-REACH(S, p)
        B ← BWD-REACH(S, p)
        parallel for vertices u ∈ F ∩ B do
            color(u) ← p
        end
        F_1 ← F_1 \ (F ∩ B)
        INSERTIFNOTEMPTY(P_2, S \ (F ∪ B)
        INSERTIFNOTEMPTY(P_2, F \ B)
        INSERTIFNOTEMPTY(P_2, B \ F)
    end
    SWAP(P_1, P_2)
    P_2 ← ∅
    fb ← fb + 1
end

while F_1 ≠ ∅ do
    parallel for vertices u ∈ F_1 do
        color(u) ← u
    end
    while fix-point is not reached do
        parallel for vertices u ∈ F_1 do
            FWD-MAXCOLOR(u, color)
        end
    end
    P, S ← PIVOTWITHSET(colors)
    F_2 ← ∅
    parallel for (p_i, S_i ∈ P, S) do
        B ← BWD-REACH(S_i, p_i)
        parallel for vertices u ∈ B do
            color(u) ← p_i
            APPEND(F_2, u)
        end
    end
    F_1 ← F_1 \ F_2
end
```

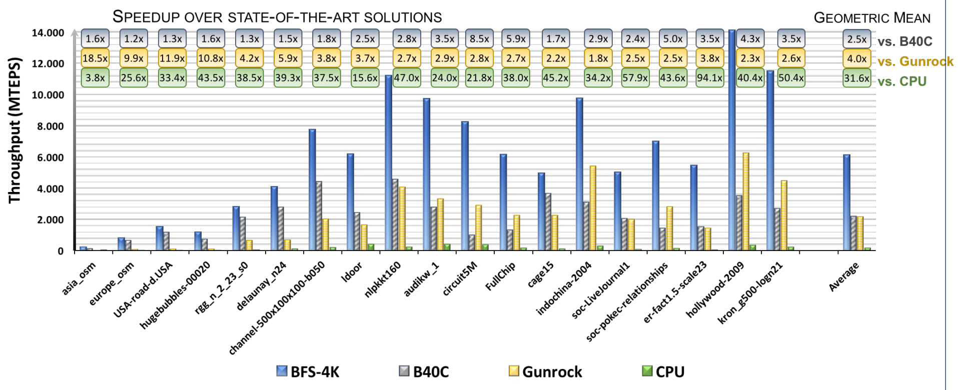

Figure 1 Performance comparison of BFS GPU implementations. Comparison between the BFS implementation adopted in this work[1] and the other most widespread BFS applications for GPUs at the state of the art.

Figures 2, 3 and 4 of the main paper. Figures 16, 17 and 18 report the performance of *cuRnet* measured by running the software on two different GPU architectures. Regarding BFS (Figure 16), the device with the Maxwell architecture outperforms the Tesla device, however also the less recent device shows good speed-ups, up to 10x, w.r.t. iGraph. Test concerning the SCC search show similar results, with some exception in which the Tesla architecture outperforms the Maxwell one due to the very small workloads (small homology networks), as shown in Figure 18. Tests over PPI networks (see Figure 17), regarding the calculation of distance in shortest paths, show that the architectural difference between the two devices may results in different slopes of the running times that produce differences in speed-up curves. Finally, Figure 19 reports the PCSF subnetworks obtained by running PCSF accelerated with the *cuRnet* SSSP function to analyze diffuse large B-cell lymphoma (DLBCL). Based on gene expression profiling studies DLBCL can be divided into two subgroups, the germinal center B-cell (GCB) and the activated B-cell like (ABC), with different clinical outcome and response to therapies [11, 12]. In particular, Figure 19 shows subnetworks for GCB patients. It highlights the activation of the PI3K/Akt/mTOR signalling pathway (cluster in red) and over-expression of germinal center markers such as BCL6, LMO2, MME (CD10) and MYBL1m, confirming the findings reported in [13, 14].

Author details
[1]Department of Computer Science, Strada le Grazie, 15, Italy, Verona, Italy. [2]Institute of Oncology Research (IOR), Via Vincenzo Vela 6, Bellinzona, Switzerland.

References

1. Busato, F., Bombieri, N.: BFS-4K: an efficient implementation of BFS for kepler GPU architectures. IEEE Trans. Parallel Distrib. Syst. **26**(7), 1826–1838 (2015)
2. Wang, Y., Davidson, A., Pan, Y., Wu, Y., Riffel, A., Owens, J.D.: Gunrock: A high-performance graph processing library on the GPU. In: Proc. ACM PPoPP, pp. 265–266 (2016)
3. Merrill, D., Garland, M., Grimshaw, A.: Scalable GPU graph traversal. In: Proc. of ACM PPoPP, pp. 117–128 (2012)
4. Davis, T.A., Hu, Y.: The University of Florida sparse matrix collection. ACM Transactions on Mathematical Software **38**(1), 1 (2011)
5. Bader, D.A., Meyerhenke, H., Sanders, P., Wagner, D.: Graph partitioning and graph clustering, 10th DIMACS implementation challenge workshop. Contemporary Mathematics **588** (2013)
6. Leskovec, J., et al.: Stanford network analysis project (2010)
7. Tao, G., Yutong, L., Guang, S.: Using MIC to Accelerate a Typical Data-intensive Application: The Breadth-first Search, pp. 1117–1125 (2013)
8. Busato, F., Bombieri, N.: "An Efficient Implementation of the Bellman-Ford Algorithm for Kepler GPU Architectures". IEEE Transactions on Parallel and Distributed System **27**(8), 2222–2223 (2016)
9. Aldegheri, S., Barnat, J., Bombieri, N., Busato, F., Ceska, M.: Parametric multi-step scheme for gpu-accelerated graph decomposition into strongly connected components. In: Euro-Par 2016: Parallel Processing Workshops - Euro-Par 2016 International Workshops, Grenoble, France, August 24-26, 2016, Revised Selected Papers, pp. 519–531 (2016)
10. Franceschini, A., *et al.*: "string v9. 1: protein-protein interaction networks, with increased coverage and integration". Nucleic acids research **41**(D1), 808–815 (2012)
11. Testoni, M., Zucca, E., Young, K., Bertoni, F.: Genetic lesions in diffuse large b-cell lymphomas. Annals of Oncology **26**(6), 1069–1080 (2015)
12. Dalla-Favera, R.: Molecular genetics of aggressive b-cell lymphoma. Hematological Oncology **35**(S1), 76–79 (2017)
13. Roschewski, M., Staudt, L.M., Wilson, W.H.: Diffuse large b-cell lymphoma [mdash] treatment approaches in the molecular era. Nature reviews Clinical oncology **11**(1), 12–23 (2014)
14. Pon, J.R., Marra, M.A.: Clinical impact of molecular features in diffuse large b-cell lymphoma and follicular lymphoma. Blood **127**(2), 181–186 (2016)

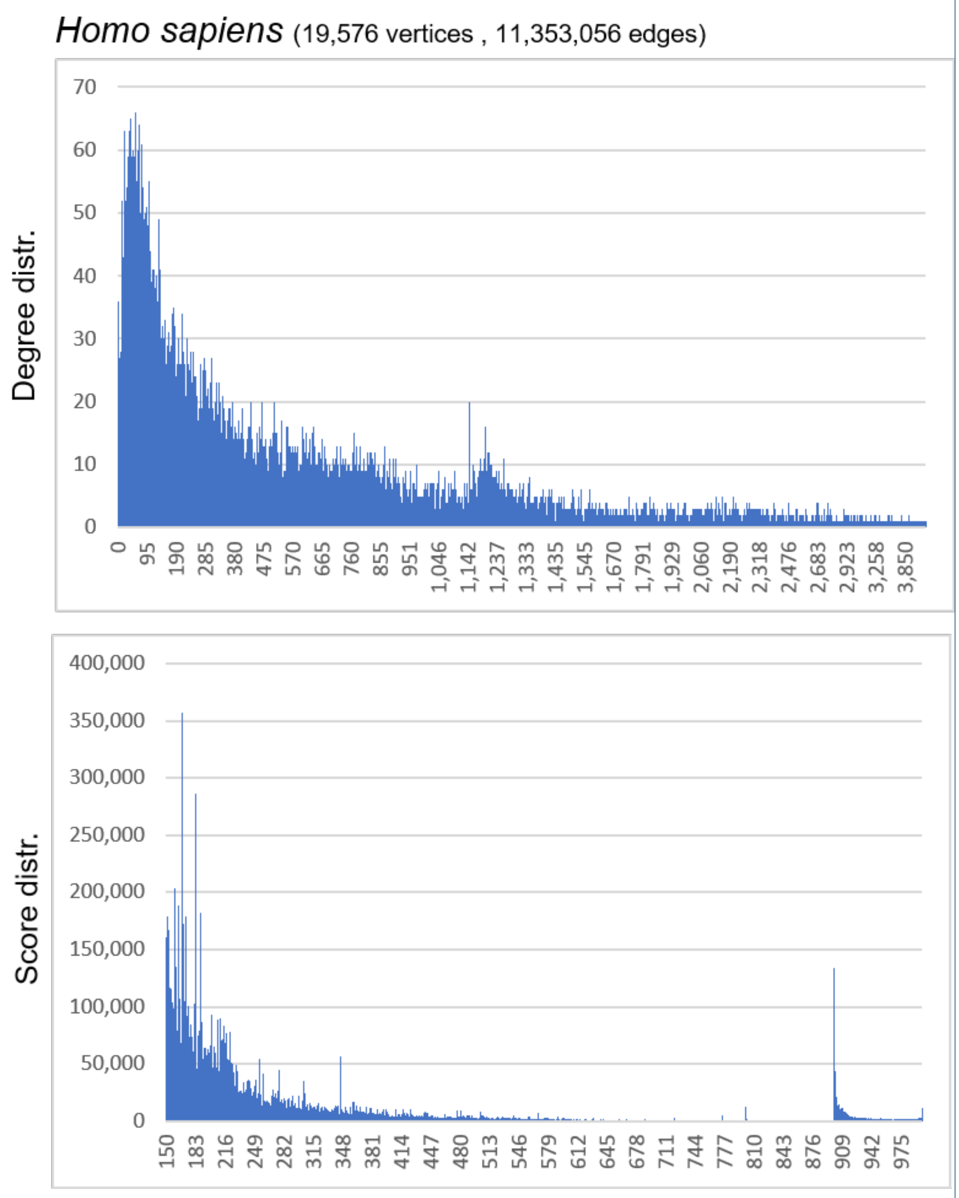

Figure 2 Properties of benchmark *Homo sapiens* PPI obtained from STRING. Number of vertices, number of edges, vertices degree distribution and edges weights distribution for the three PPI networks downloaded via the R package of STRINGdb.

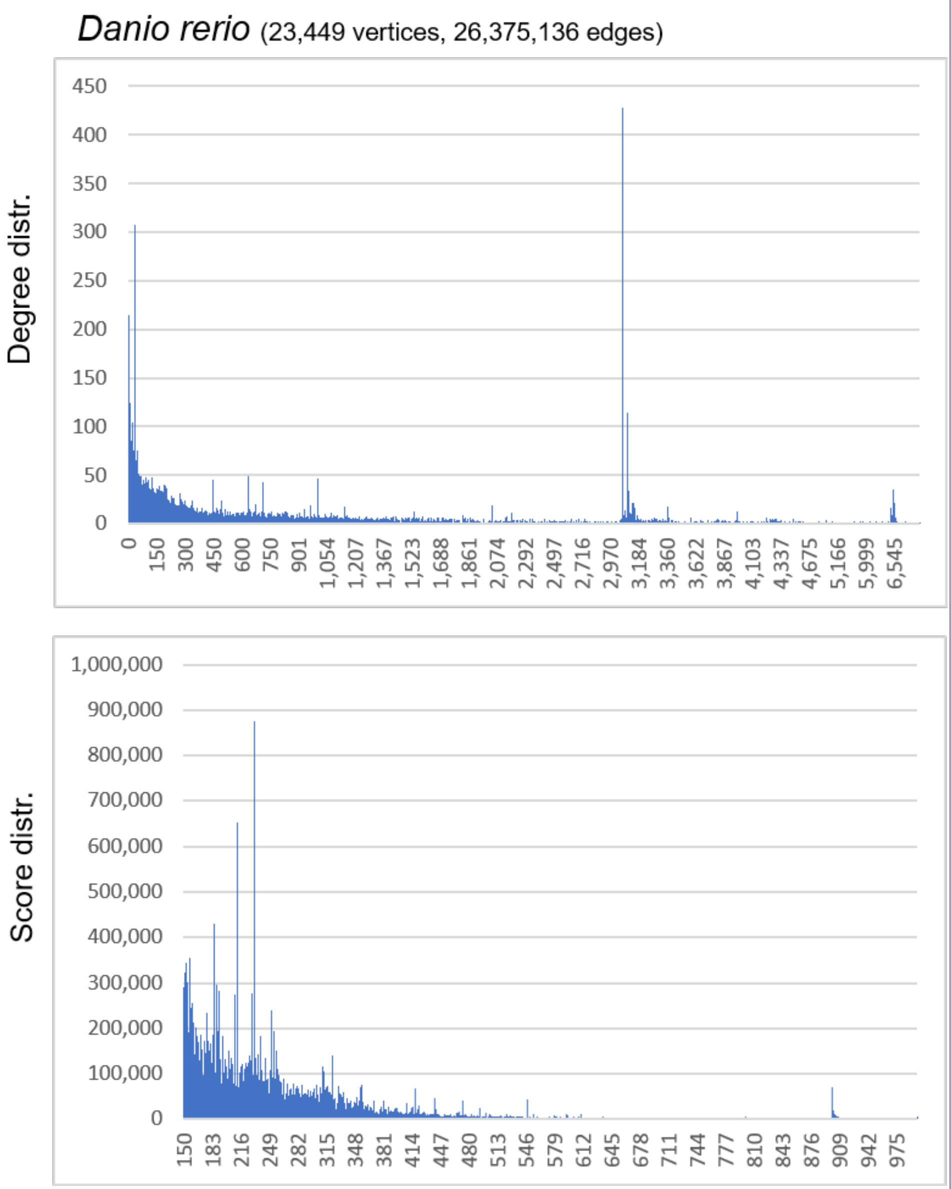

Figure 3 Properties of benchmark *Danio rerio* **PPI obtained from STRING.** Number of vertices, number of edges, vertices degree distribution and edges weights distribution for the three PPI networks downloaded via the R package of STRINGdb.

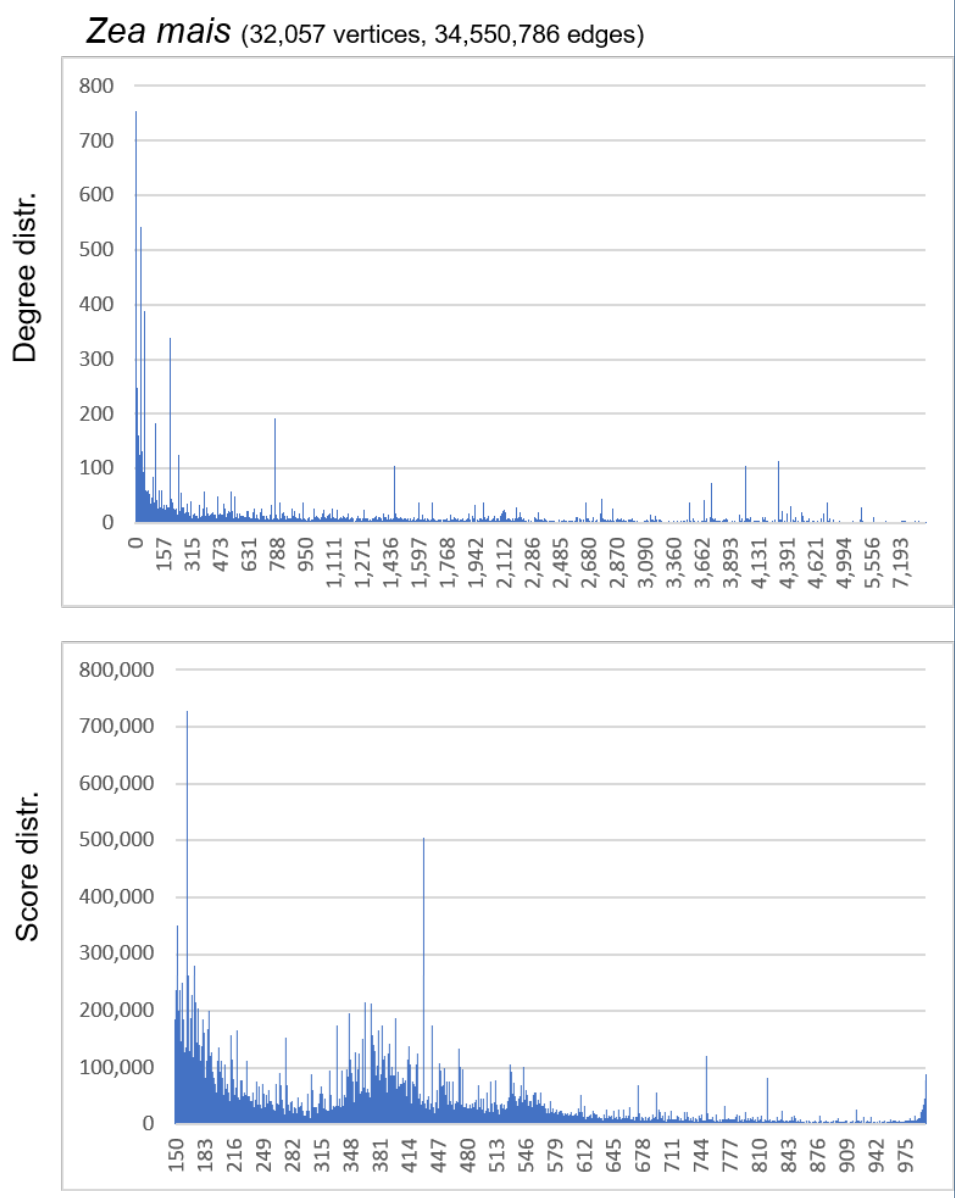

Figure 4 Properties of benchmark $Zea\ mais$ **PPI obtained from STRING.** Number of vertices, number of edges, vertices degree distribution and edges weights distribution for the three PPI networks downloaded via the R package of STRINGdb.

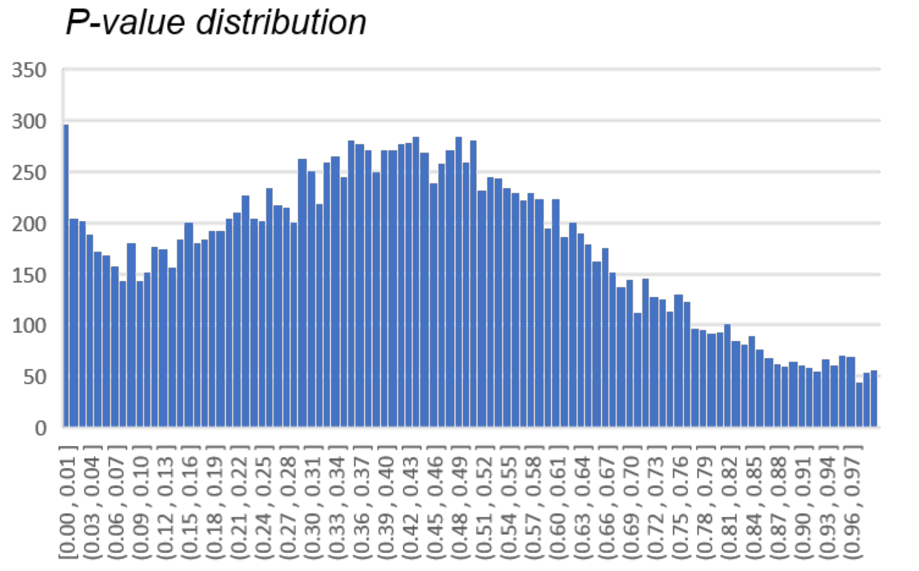

Figure 5 Distribution of p-values, over the complete $Homo\ sapiens$ **PPI**. Values were obtained from the STRINGdb R package example regarding treatments of lung cancer. Among the $17,740$ annotated genes, $1,063$ of them show a p-value below 0.05.

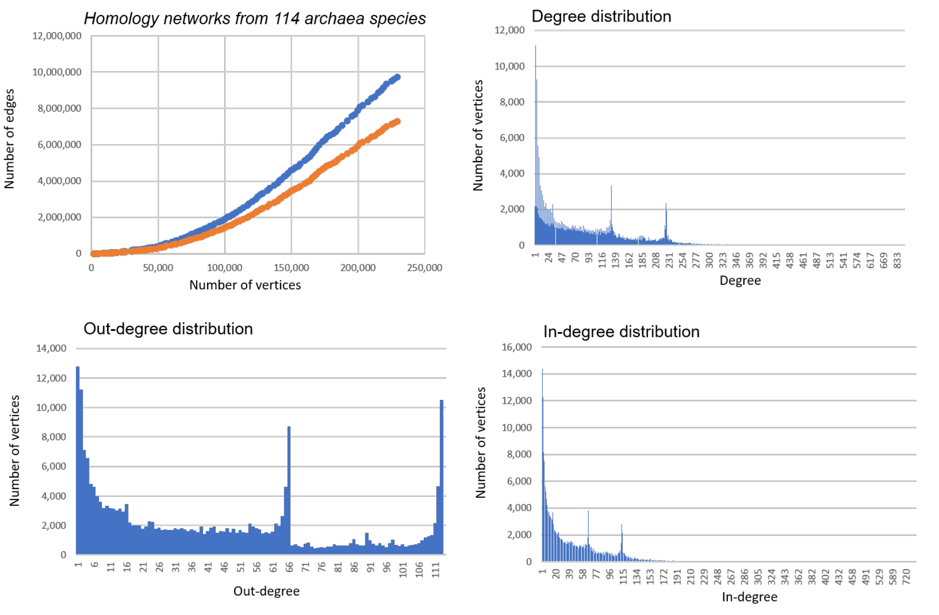

Figure 6 Properties of benchmark regarding homology networks retrieved from STRING. The networks were built by incrementally taking into account the 115 archaea species reported in STRING. Network edges connect most similar proteins among species. The top-left chart shows the number of directed edges (overlying blue line) and bidirectional edges (underlying orange line) arranged by number of vertices of the incremental networks. The ratio between the two type of edges ranges between 74 and 76 percent. The final complete network has $229,363$ vertices and $9,725,349$ edges. In, out, and undirected degree distribution of the complete network are show in the remaining three charts. The maximum out degree is 114, while undirected degree and in degree reach values of about $3,500$. Distributions shows intermediate peaks at 132 and 226 for undirected degree, 66 and 113 for out and in degree. The complete network has $3,643,915$ bidirectional edges. The degree distribution of bidirectional edges (not shown) follows a trend similar tot he out degree distribution. Average degrees are 84, 43, 42 and 33 for undirected, in, out and bidirectional edges, respectively.

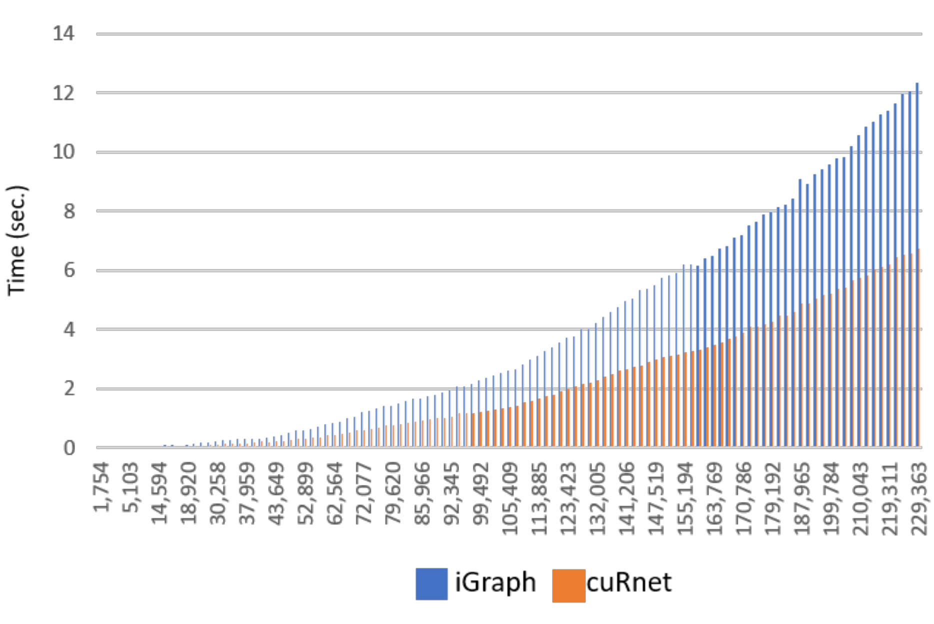

Figure 7 Timing for building graph data structures in PPI dataset.

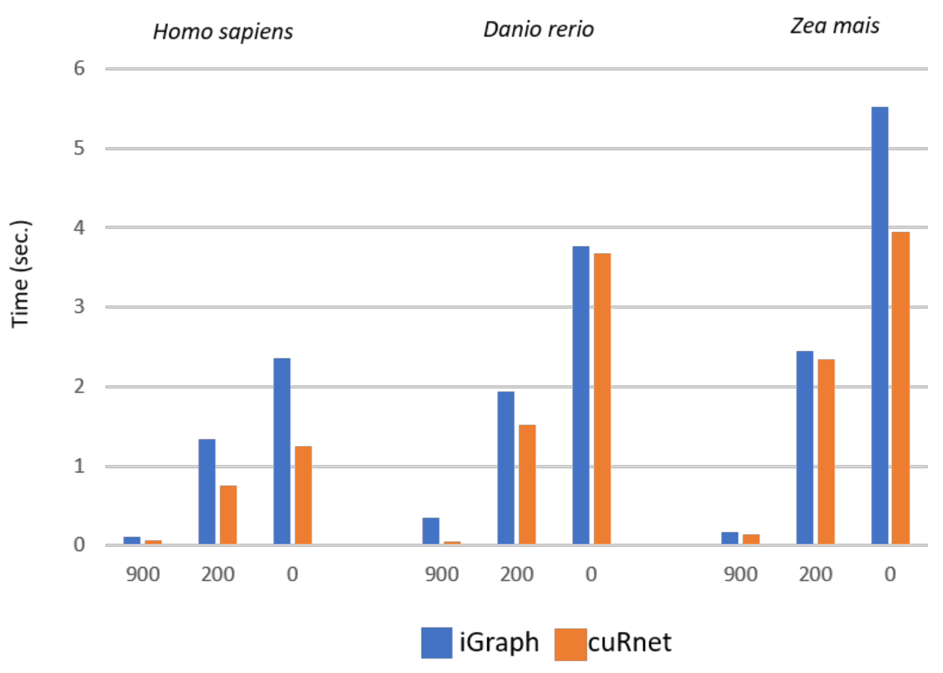

Figure 8 Timing for building graph data structures in homology networks.

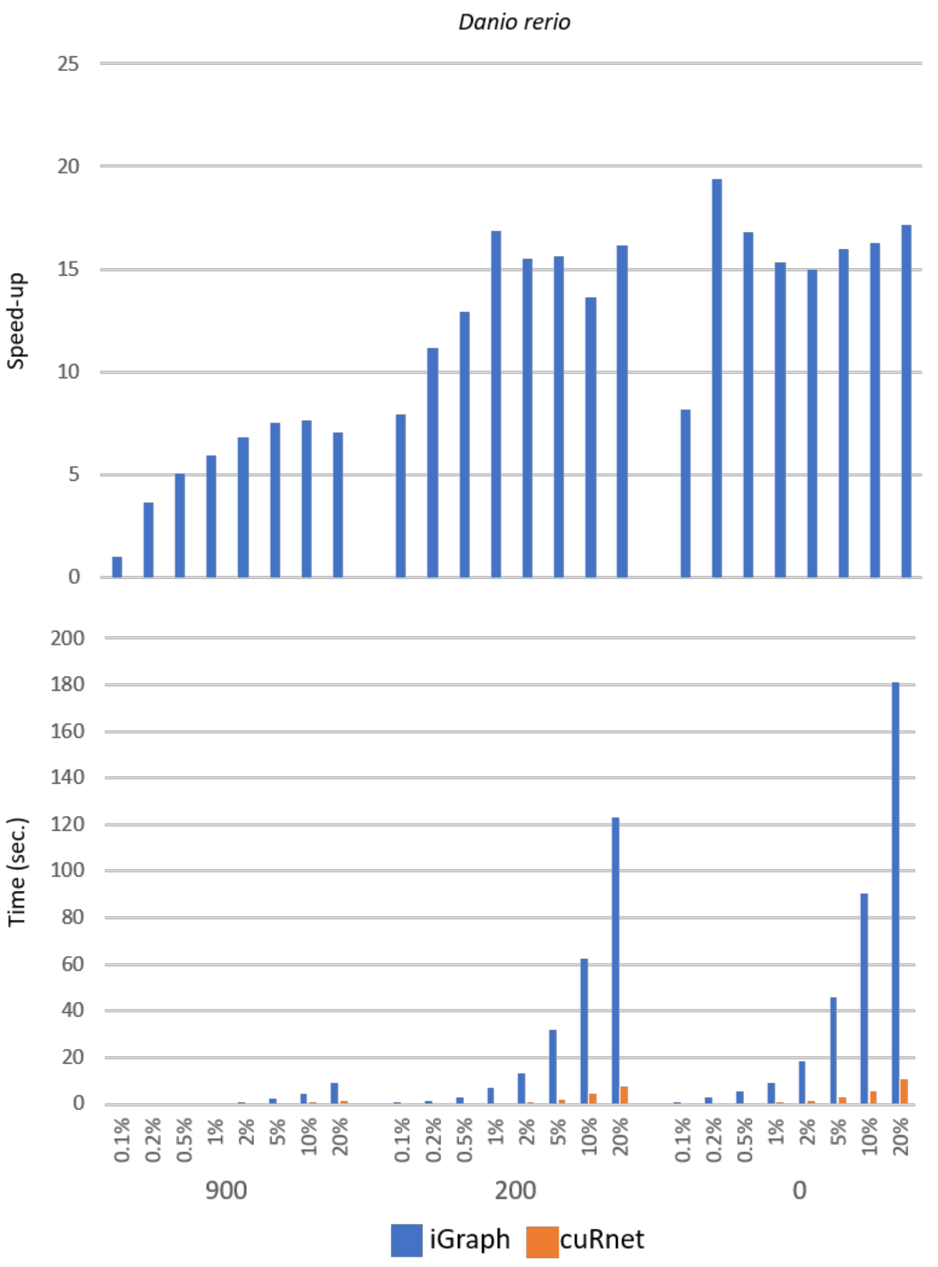

Figure 9 cuRnet performance vs iGraph on computing breath first search on the *Danio rerio* PPI. Three different score thresholds, 0, 200 and 900, were applied, and different amounts of source vertices were selected.

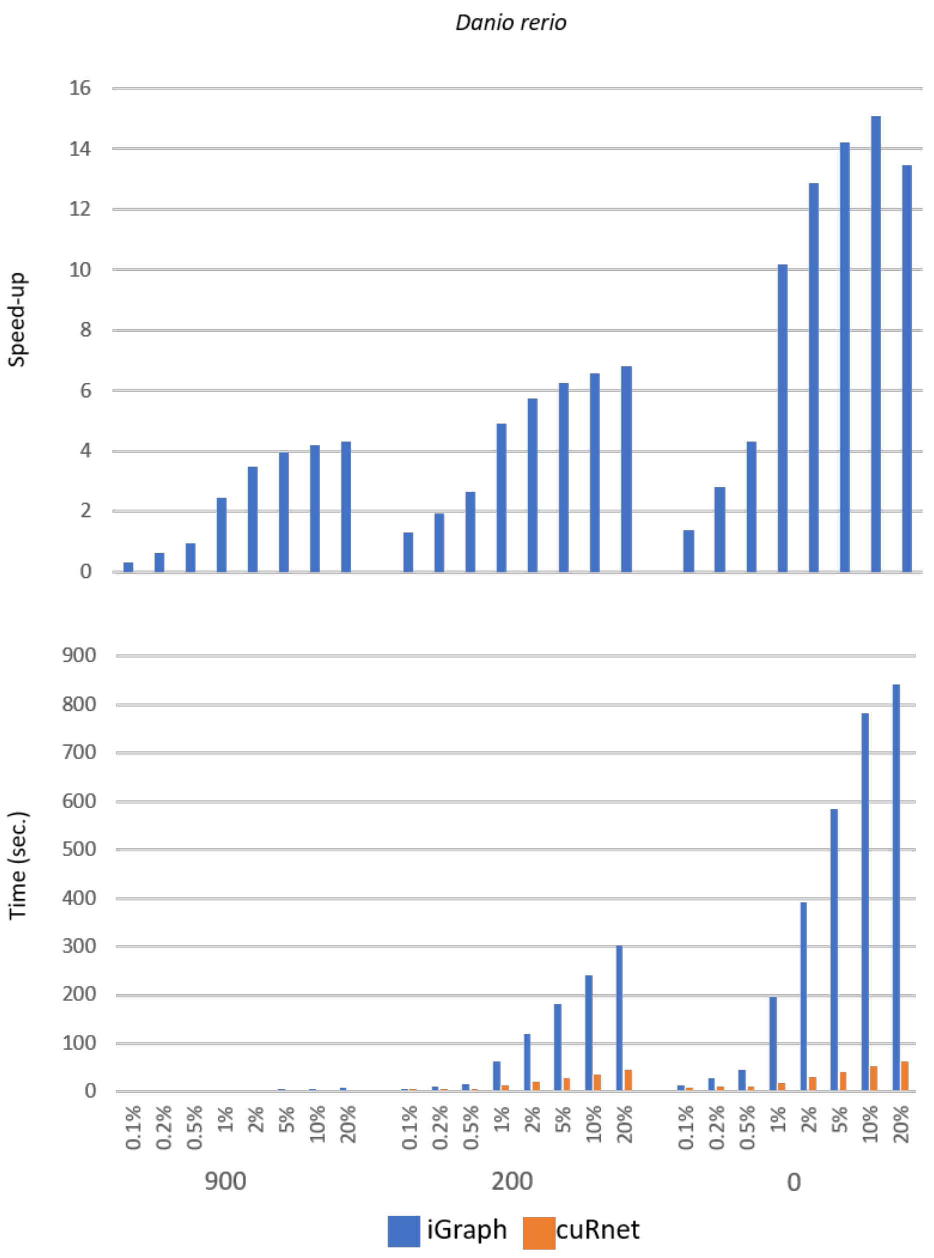

Figure 10 cuRnet performance vs iGraph on computing shortest paths distances on the $Danio\ rerio$ **PPI.** Three different score thresholds, 0, 200 and 900, were applied, and different amounts of source vertices were selected. The underlying charts show running times of *cuRnet* and iGraph in calculating distance of shortest paths within the PPI of the selected species for every combination of score threshold and amount of selected sources.

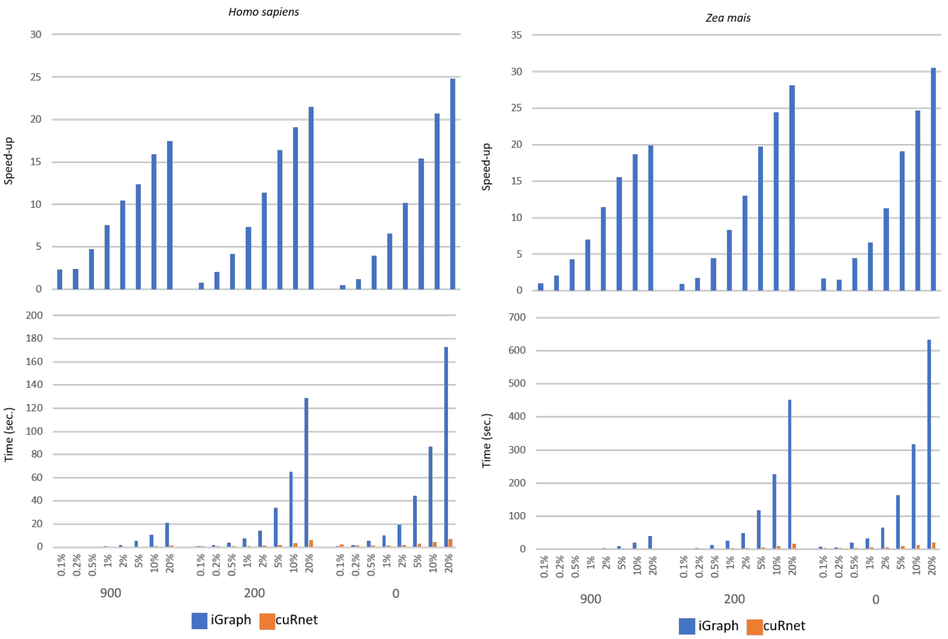

Figure 11 cuRnet performance vs iGraph on computing Breath First Search on the *Homo sapiens* **and** *Zea mais* **PPIs.** Timing includes the graph data structure computation. Algorithms are run on unlabelled PPI datasets.

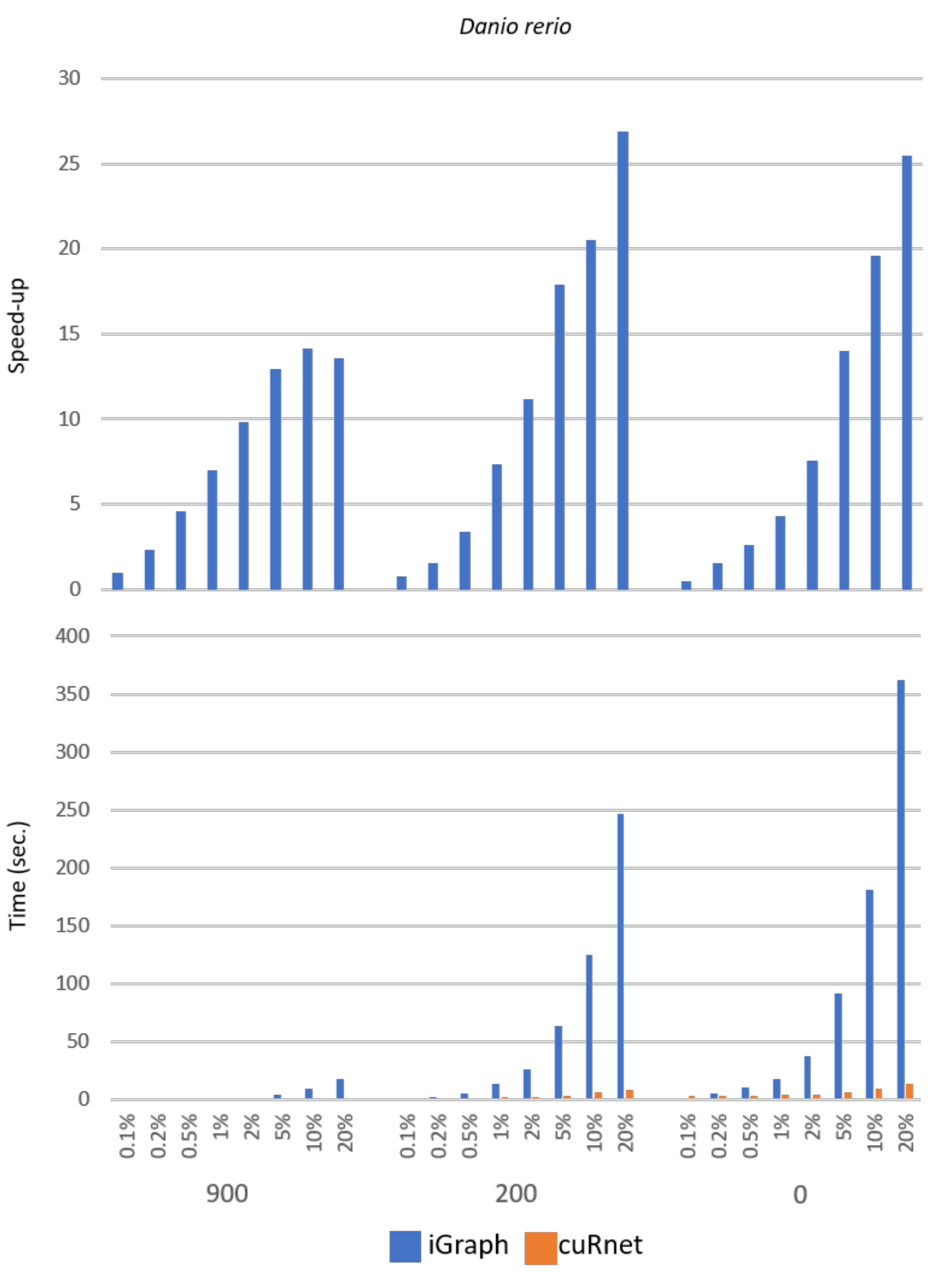

Figure 12 cuRnet performance vs iGraph on computing Breath First Search on the $\textit{Danio rerio}$ **PPI.** Timing includes the graph data structure computation. Algorithms are run on unlabelled PPI datasets.

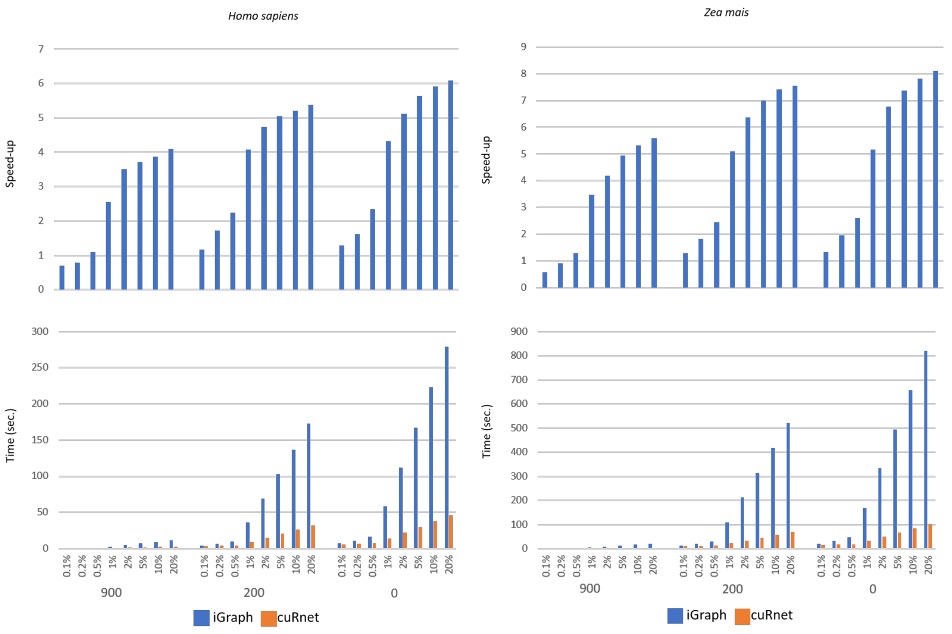

Figure 13 cuRnet performance vs iGraph on computing shortest paths distances on the *Homo sapiens* and *Zea mais* PPIs. Timing includes the graph data structure computation. Algorithms are run on labeled PPI datasets.

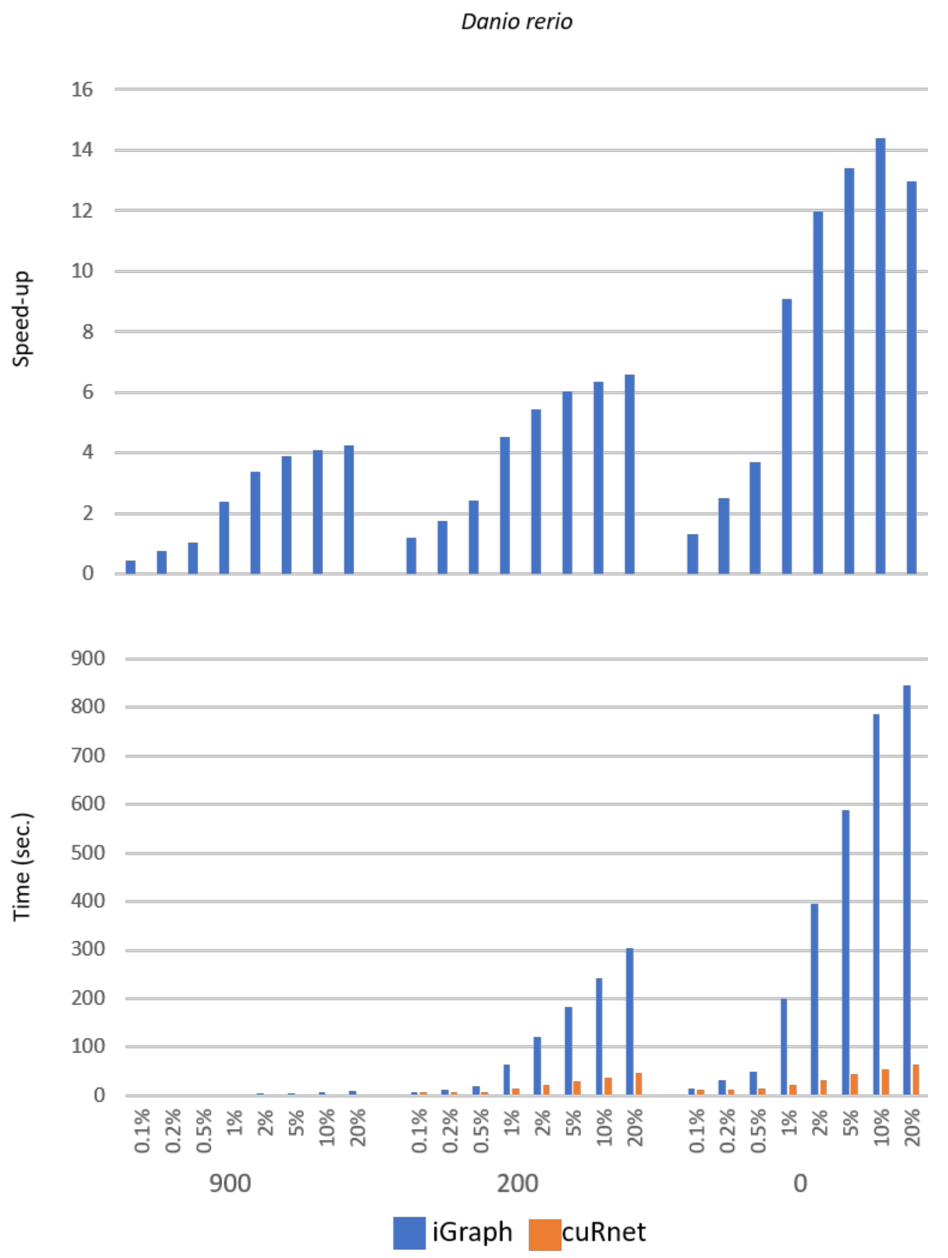

Figure 14 cuRnet performance vs iGraph on computing shortest paths distanceson the *Danio rerio* **PPI.** Timing includes the graph data structure computation. Algorithms are run on labeled PPI datasets.

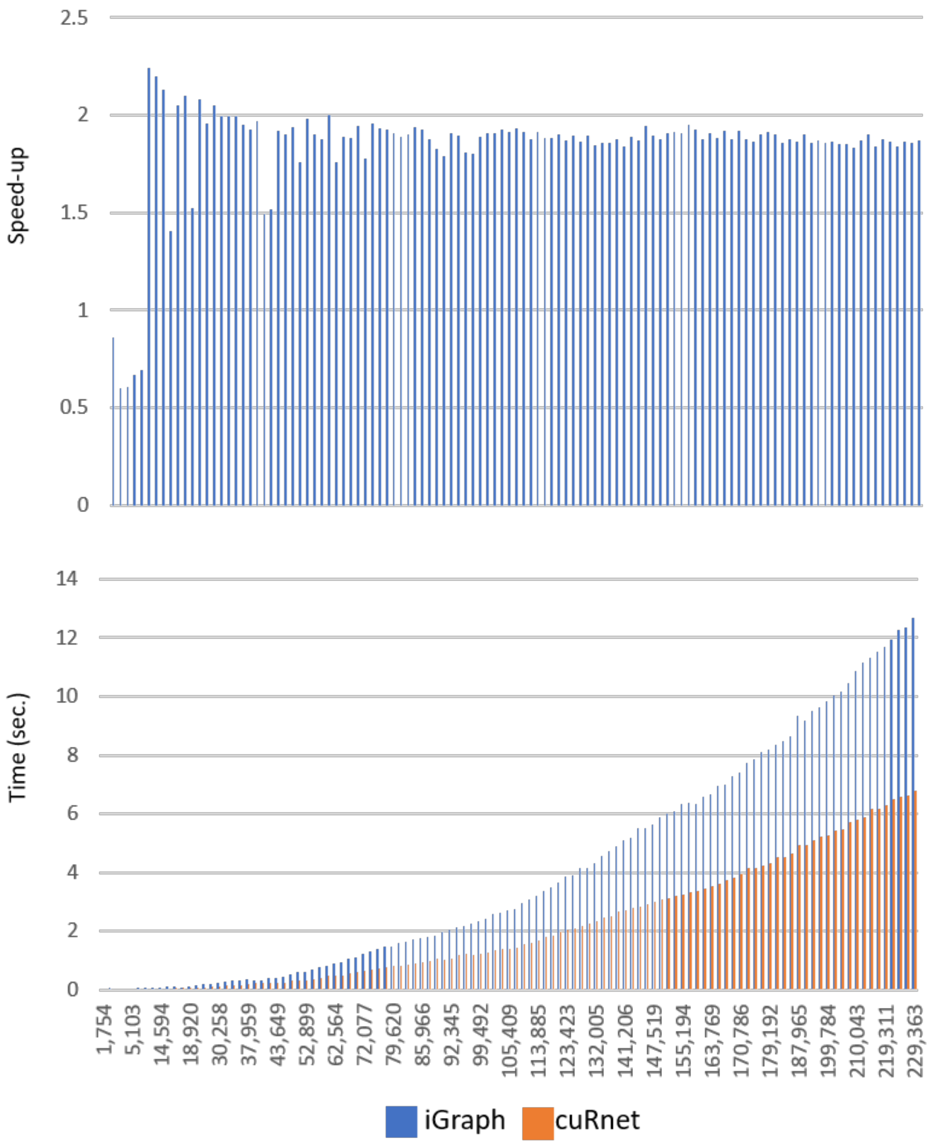

Figure 15 cuRnet performance vs iGraph on computing strongly connected components. . Timing includes the graph data structure computation. Algorithms are run on homology directed unlabelled networks.

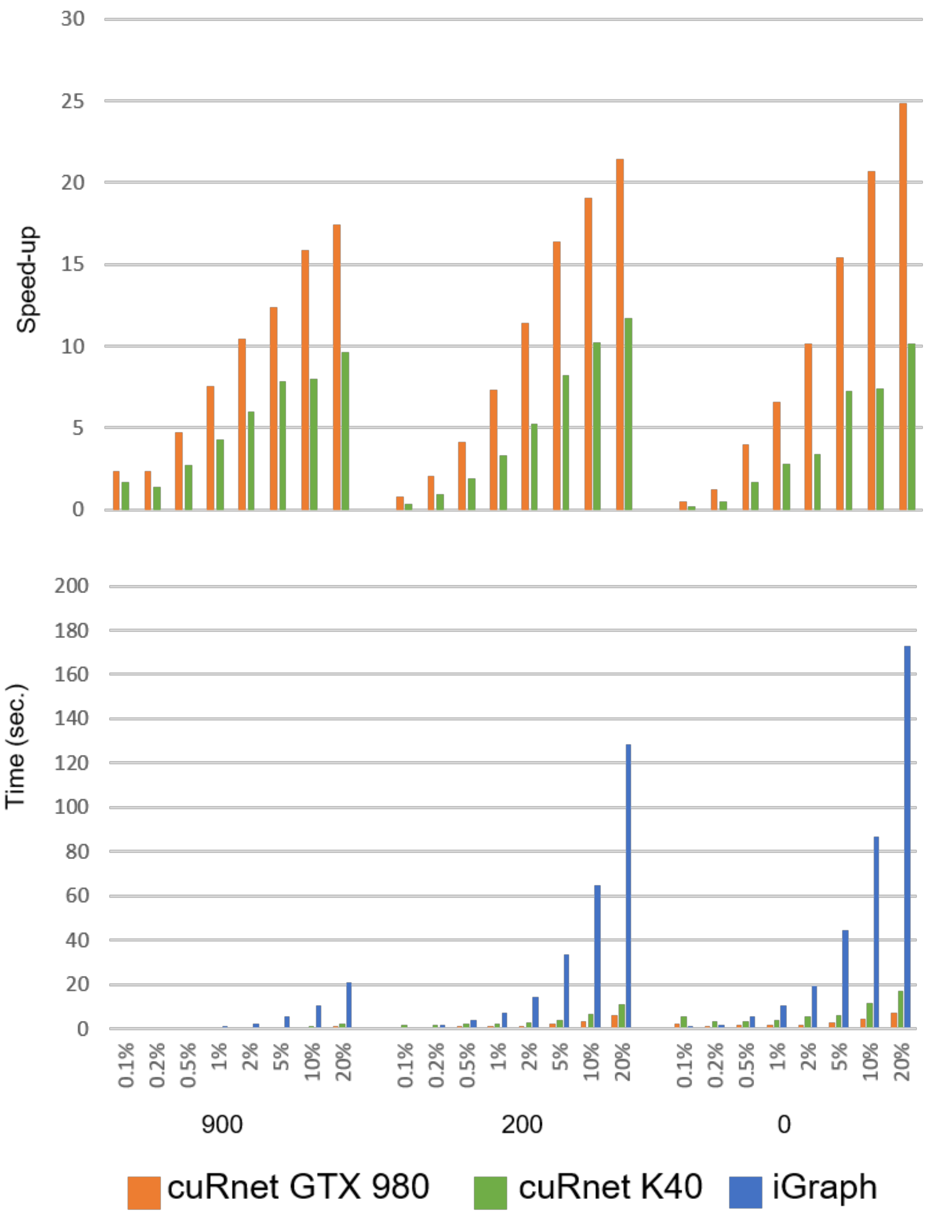

Figure 16 cuRnet performances on computing Breath First Search by running the software on two differnt NVIDIA GPU devices, the Maxwell GeForce GTX 980 and the Tesla K40.. Timing includes the graph data structure computation. Algorithms are run on unlabelled PPI datasets.

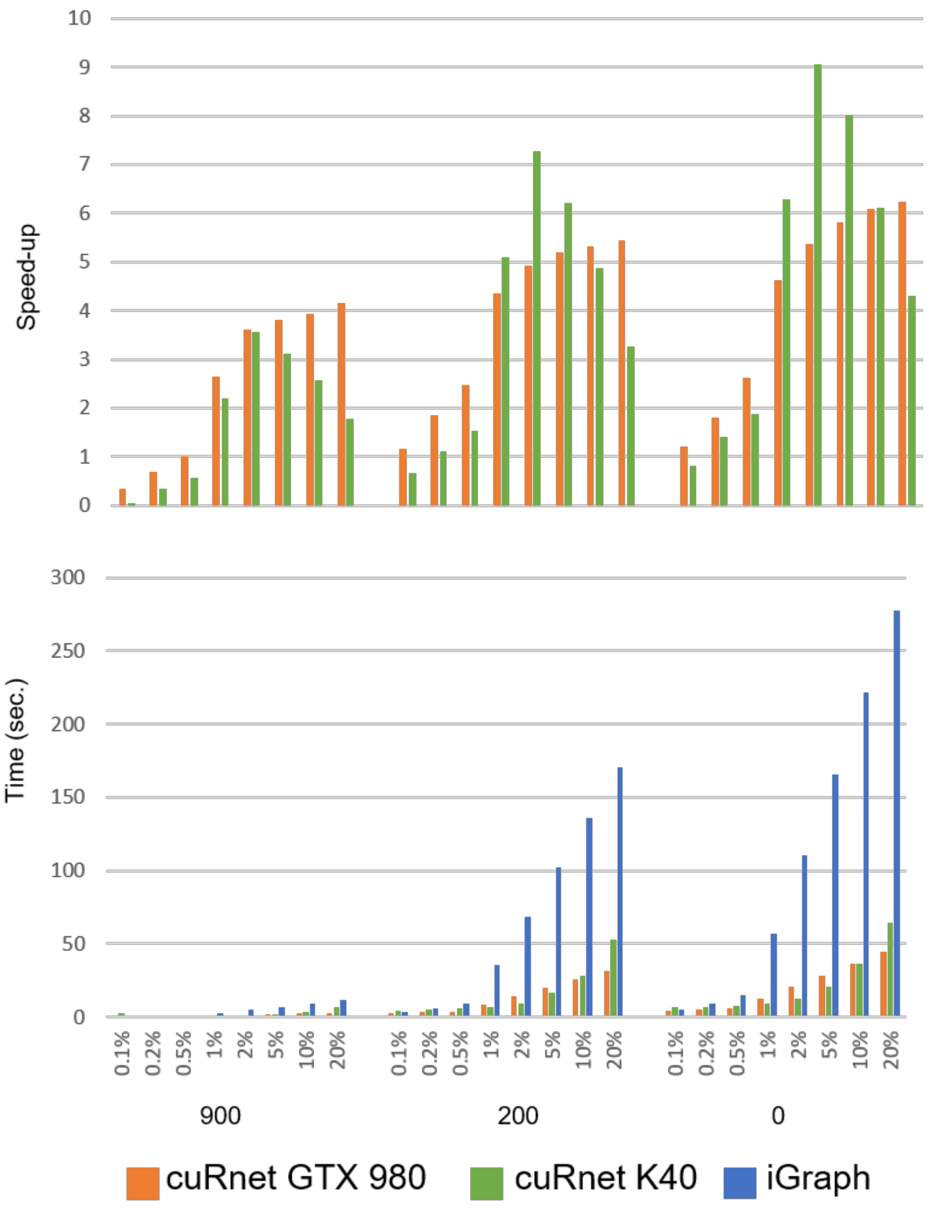

Figure 17 cuRnet performances on computing shortest paths distances by running the software on two differnt NVIDIA GPU devices, the Maxwell GeForce GTX 980 and the Tesla K40.. The underlying charts show running times of the executions of *cuRnet* on the two devices, and their speed-ups w.r.t. iGraph, in calculating distance of shortest paths within the PPI of the Homo sapiens species for every combination of score threshold and amount of selected sources.

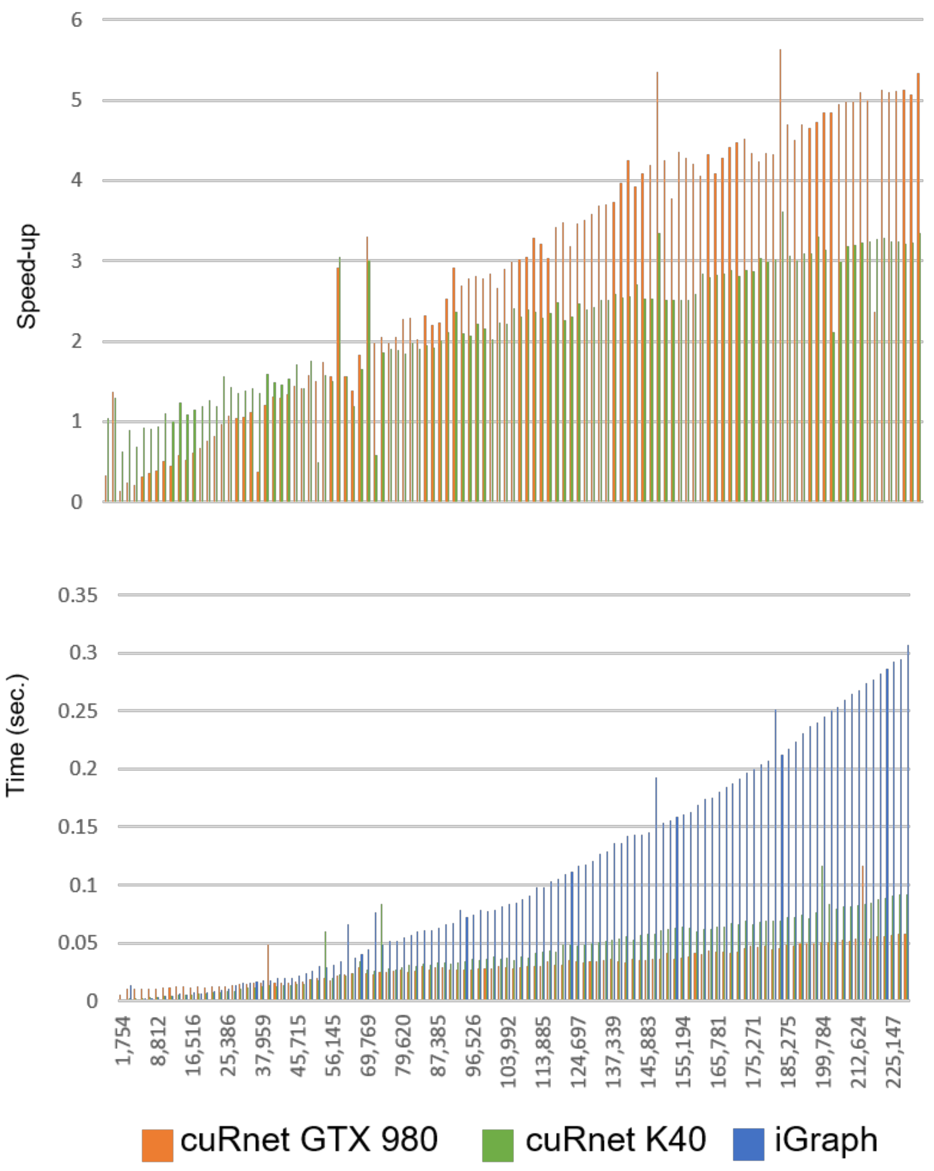

Figure 18 cuRnet performances on computing Breath First Search by running the software on two differnt NVIDIA GPU devices, the Maxwell GeForce GTX 980 and the Tesla K40.. Running times, and corresponding speed-ups, of *cuRnet* on the two devices, and their speed-ups w.r.t. iGraph, on increasing the size of the extracted homology network, up to the final one of 114 species.

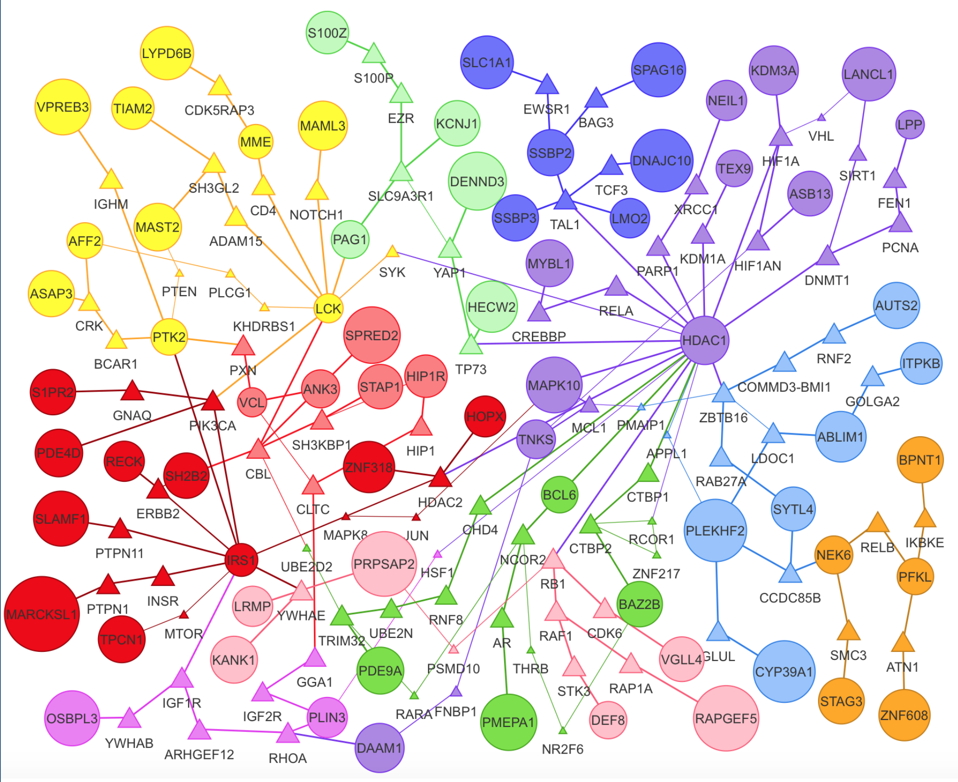

Figure 19 PCSF accelerate with cuRnet SSSP function on GCB patients.
